# High-Dose Vitamin D_3_ Supplementation During Pregnancy and Test-Based Cognitive Performance at Age 10 Years: A Post Hoc Secondary Analysis of a Randomized Clinical Trial

**DOI:** 10.1001/jamanetworkopen.2026.11464

**Published:** 2026-05-18

**Authors:** Olivia Frigast Frederiksen, Jens Richardt Møllegaard Jepsen, Nicklas Brustad, Rebecca Vinding, Julie Bøjstrup Rosenberg, Parisa Mohammadzadeh, María Hernández-Lorca, Ann-Marie Malby Schoos, Nilo Vahman, Birte Y. Glenthøj, Birgitte Fagerlund, Niels Bilenberg, Klaus Bønnelykke, Bjørn H. Ebdrup, Kristina Aagaard, Bo Chawes

**Affiliations:** 1 Copenhagen Prospective Studies on Asthma in Childhood, Herlev and Gentofte Hospital, University of Copenhagen, Copenhagen, Denmark; 2 Center for Neuropsychiatric Schizophrenia Research, Mental Health Center, Glostrup, Copenhagen University Hospital–Mental Health Services CPH, Copenhagen, Denmark; 3 Child and Adolescent Mental Health Center, Copenhagen University Hospital–Mental Health Services CPH, Copenhagen, Denmark; 4 Department of Pediatrics and Adolescent Medicine, Copenhagen University Hospital–Herlev and Gentofte, Herlev, Denmark; 5 Department of Clinical Medicine, Faculty of Health and Medical Sciences, University of Copenhagen, Copenhagen, Denmark; 6 Department of Pediatrics, Copenhagen University Hospital–Næstved, Slagelse and Ringsted, Slagelse, Denmark; 7 Department of Pediatrics, Copenhagen University Hospital–Amager and Hvidovre, Hvidovre, Denmark; 8 Department of Psychology, University of Copenhagen, Copenhagen, Denmark; 9 Institute of Clinical Research, University of Southern Denmark, Odense, Denmark

## Abstract

**Importance:**

Observational studies have reported associations between pregnancy vitamin D levels and offspring cognition, but no randomized clinical trial (RCT) has investigated the effect of high-dose vitamin D_3_ supplementation in pregnancy on offspring cognition into middle childhood.

**Objective:**

To assess whether high-dose vitamin D_3_ supplementation during pregnancy is associated with improved offspring cognition at age 10 years.

**Design, Setting, and Participants:**

This is a post hoc secondary analysis of the blinded, placebo-controlled Copenhagen Prospective Studies on Asthma in Childhood 2010 RCT conducted from March 4, 2009, to November 17, 2010. The cohort included 700 mother-child pairs from Denmark, of whom 623 were randomized. Exclusion criteria were vitamin D intake above 600 IU/d; endocrine, kidney, or heart disease; and insufficient Danish language proficiency. Cognitive assessments were conducted for the offspring at age 10 years, excluding those born at less than 28 weeks’ gestation and with birth weight less than 1500 g. Participants were unblinded at 3 years of age, while investigators remained unaware of group assignments. The 10-year visits were conducted from February 11, 2019, to December 13, 2021. Statistical analyses were conducted from February to June 2025.

**Intervention:**

High-dose (2800 IU/d) or standard-dose (400 IU/d) vitamin D_3_ from pregnancy week 24 to 1 week post partum.

**Main Outcome and Measures:**

The primary outcome was cognitive functioning across 11 functions assessed at age 10 years using a comprehensive neuropsychological test battery as part of the Copenhagen Prospective Study on Neuro-Psychiatric Development (COPSYCH).

**Results:**

The vitamin D_3_ RCT and the COPSYCH visit were completed by 498 children (mean [SD] age, 10.3 [0.4] years; 258 boys [51.8%]): 247 prenatally exposed to high-dose vitamin D_3_ and 251 prenatally exposed to standard-dose vitamin D_3_. Covariate-adjusted analyses of standardized scores (mean [SD], 0 [1]; higher values indicating better performance) showed positive associations of high-dose vitamin D_3_ with verbal memory (β = 0.17 SD; 95% CI, 0.03-0.32 SD; *P* = .02), visual memory (β = 0.24 SD; 95% CI, 0.06-0.42 SD; *P* = .01), and flexibility or set shift (β = 0.19 SD; 95% CI, 0.01-0.37 SD; *P* = .04); however, high-dose vitamin D_3_ was no longer associated with flexibility or set shift after multiple test correction.

**Conclusion and Relevance:**

This post hoc analysis of an RCT suggests that high-dose vitamin D_3_ supplementation in pregnancy is positively associated with visual memory, verbal memory, and flexibility or set shift among offspring measured at age 10 years. These findings strengthen evidence on the association of prenatal vitamin D exposure with childhood cognition.

**Trial Registration:**

ClinicalTrials.gov Identifier: NCT00856947

## Key Points

**Question:**

Is high-dose vitamin D_3_ supplementation during pregnancy associated with improved cognition among offspring?

**Findings:**

In this post hoc analysis of a randomized clinical trial including 498 children, high-dose vitamin D_3_ supplementation was associated with better verbal and visual memory and set shift at age 10 years compared with standard-dose vitamin D_3_, although the association with flexibility or set shift did not remain significant after false discovery rate correction.

**Meaning:**

This study suggests that high-dose vitamin D_3_ supplementation during pregnancy may be associated with improved cognitive functioning at age 10 years.

## Teaser

This post hoc secondary analysis of a randomized clinical trial assesses whether high-dose vitamin D_3_ supplementation during pregnancy is associated with improved offspring cognition at age 10 years compared with standard-dose vitamin D_3_ supplementation.

## Introduction

Childhood cognition is a predictor of socioeconomic status, occupational achievement, and cognitive abilities later in life.^1,2,3,4^ Although heritability estimates for cognition are reported as high as 80%,^5^ meta-analyses show that several prenatal exposures can be associated with worse cognitive outcomes,^6,7^ illustrating how early environmental factors may shape cognitive development.

Globally, vitamin D deficiency is a widespread problem among pregnant women.^8^ Vitamin D contributes to brain development during pregnancy,^9^ with in vitro and rodent models highlighting its involvement in essential neurodevelopmental processes, including neuronal differentiation, neurotransmitter synthesis, intracellular calcium signaling, and antioxidant activity.^10,11^ The distribution of vitamin D receptors and 1-α-hydroxylase in the human brain further supports its important role in neurodevelopment.^12^ Moreover, experimental rat models have demonstrated associations between vitamin D deficiency and cognitive functions, including learning and memory impairments.^13,14^

Prenatal vitamin D deficiency has been associated with neuropsychiatric disorders such as autism spectrum disorder (ASD),^15,16^ attention-deficit/hyperactivity disorder (ADHD),^17,18^ and schizophrenia.^19^ These disorders are associated with impairments in several cognitive domains, including attention and executive functioning.^20,21,22^

Two previous studies have examined the association of prenatal vitamin D supplementation with neurodevelopment in the Copenhagen Prospective Studies on Asthma in Childhood 2010 (COPSAC_2010_) cohort; Sass et al^23^ found no effect on offspring neurodevelopment from birth to 6 years of age, and Aagaard et al^24^ reported no effect on neurodevelopmental disorders at age 10 years, but hitherto we have not investigated a potential effect on cognitive function at this age.

Studies examining gestational vitamin D levels and cognitive functioning among offspring have reported inconsistent findings. Existing observational studies differ in exposure and outcome measurements. Exposure measurements have varied between first,^25,26,27,28,29,30^ second,^25,30,31,32,33,34,35^ and third trimester^25,29,30,31,36,37,38,39,40^ as well as cord blood^25,30,31,32,34,36,41,42,43,44^ levels of 25(OH)D. Outcome measurements varied in age at testing, cognitive function assessed, and types of tests used. Positive associations with vitamin D have been reported for language skills,^25,33,40,45^ gross motor skills,^25,27,44,45^ executive functioning,^28^ and intelligence.^31,46^ Two meta-analyses of observational studies found positive associations between maternal vitamin D during pregnancy and offspring cognitive abilities, as well as fewer ADHD and ASD traits.^47,48^ To date, only 1 other randomized clinical trial (RCT) has examined the role of vitamin D supplementation during pregnancy in offspring cognition. The study reported a positive effect of supplementation with 2000 IU/d of vitamin D from 12 to 16 weeks of pregnancy until delivery on receptive and expressive language at 3 to 5 years of age, although it was limited by a relatively small sample size (n = 156).^49^ To our knowledge, no RCT has investigated the effect of prenatal vitamin D supplementation on offspring cognition in middle childhood.

The original RCT^50^ investigated whether vitamin D supplementation during pregnancy reduced the risk of persistent wheeze or asthma among offspring during the first 3 years of life, which showed no protective effect. In this post hoc analysis of the RCT, we aimed to investigate the hypothesis that high-dose compared with standard-dose vitamin D_3_ supplementation during pregnancy has a positive association with test-based cognitive performance evaluated at age 10 years as part of the Copenhagen Prospective Study on Neuro-Psychiatric Development (COPSYCH) project.^51^

## Methods

The COPSYCH study consists of cognitive and psychopathological assessments of the COPSAC_2010_ cohort, comprising 700 mother-child pairs. The original trial was powered for the primary outcome of recurrent wheeze or asthma among the offspring, assessed during repeated clinical visits from birth, with the primary end point at 3 years of age.^52^ Pregnant women from Zealand, Denmark, were enrolled at 22 to 26 weeks’ gestation from March 4, 2009, to November 17, 2010. Exclusion criteria were daily intake of vitamin D of more than 600 IU; chronic heart, kidney, or endocrine disease; and insufficient Danish language proficiency. Children were followed up from birth to age 10 years with a minimum of 14 visits at the COPSAC clinic. Additional visits were conducted if the child experienced respiratory, allergy, or skin symptoms^52^ (trial protocol and statistical analysis plan in Supplement 1). This study presents a post hoc analysis of an RCT that was designed and reported according to the Consolidated Standards of Reporting Trials (CONSORT) 2010 statement. The reporting of the present analysis follows the CONSORT reporting guideline. The study complied with the Declaration of Helsinki^53^ and was approved by the Local Ethics Committee and the Danish Data Protection Agency. Both parents provided written informed consent before enrollment. We complied with recognized codes of good research practice, including the Danish Code of Conduct for Research Integrity. We complied with national and international rules on the safety and rights of patients and healthy participants, including Good Clinical Practice as defined in the EU’s Directive on Good Clinical Practice, the International Conference on Harmonisation’s good clinical practice guidelines, and the Declaration of Helsinki. We followed national and international legislation on General Data Protection Regulation, the Danish Act on Processing of Personal Data, and the practice of the Danish Data Inspectorate.

### Study Intervention

In total, 623 pregnant women were randomized on a 1:1 basis from pregnancy week 24 to 1 week post partum to receive either high-dose vitamin D_3_ supplementation of 2400 IU daily, in addition to the recommended 400 IU, or standard-dose vitamin D_3_ supplementation of 400 IU daily as advised by the Danish National Board of Health. The participants were unblinded when all children had reached 3 years of age. Adherence was determined by counting returned capsules. The intervention had a 2 × 2 factorial trial design. In addition to vitamin D_3_, the participants were randomized to receive a daily fish oil (n-3-long-chain polyunsaturated fatty acid [LCPUFA]) supplement or olive oil capsules from week 24 to 1 week post partum.^52^

### Vitamin D Measurement

Maternal 25(OH)D levels were measured at week 24 of pregnancy (before intervention) and 1 week post partum (after intervention). Child levels were measured at 6 months and 6 years of age.^24^

### Outcome Measures

The outcome was assessed with neuropsychological tests administered at the COPYSCH 10-year visit. The visit was carried out between February 11, 2019, and December 13, 2021, and extended over 2 days. Day 1 consisted of a neurocognitive and neuropsychiatric assessment, and day 2 included a brain magnetic resonance imaging scan.^51^

The neurocognitive test battery included subtests from several cognitive tests. Completion of the test battery took approximately 2 hours, with breaks provided when needed.^51^ The test battery was administered by trained professionals (J.R.M.J., J.B.R., and P.M.). Descriptions of all included tests can be found in the eMethods in Supplement 2. The cognitive domains are described in the COPSYCH protocol.^51^ We included 8 distinct cognitive domains (intelligence, processing speed, reaction time, attention, motor function, memory, working memory, and executive functions) encompassing 11 functions (Table 1).

**Table 1.** Overview of Domains, Functions, Tests, and Outcome Metrics in the Neurocognitive Test Battery

Domain and function	Test	Outcome metric
Intelligence		
Estimated intelligence	Vocabulary (WISC-IV)	Total No. correct
	Matrices (WICV-IV)	Total No. correct
Processing speed		
Speed of processing	Coding (WISC-IV)	Total No. correct
	Symbol search (WISC-IV)	Sum of total No. correct, with errors subtracted
Reaction time		
Reaction time	Reaction time (CANTAB)	Simple- and 5-choice reaction time
Attention		
Sustained attention	Rapid visual information processing (CANTAB)	A-prime (unitless sensitivity score)
Motor function		
Motor speed	Reaction time (CANTAB)	Simple- and 5-choice movement time
Memory		
Verbal memory	Word selective reminding–immediate recall and object recall (TOMAL 2)	Total No. of words recalled over 6 learning trials and total No. of objects recalled over 5 learning trials
Visual memory	Paired associates learning (CANTAB)	Total errors (adjusted)
Working memory		
Verbal working memory	Digit span and letter-number sequencing (WISC-IV)	Total No. of correct forward and backward sequences and total No. of correct sequences
Executive function		
Flexibility or set shift	Intra-extra dimensional set shift (CANTAB)	Extra-dimensional stage errors
Spatial working memory	Spatial working memory (CANTAB)	Total No. of errors
Planning	Stockings of Cambridge (CANTAB)	Problems solved in minimum moves

Abbreviations: CANTAB, Cambridge Neuropsychological Test Automated Battery; TOMAL 2, Test of Memory and Learning–second edition; WISC-IV, Wechsler Intelligence Scale for Children–fourth edition.

Seven subtests from the Cambridge Neuropsychological Test Automated Battery (CANTAB)^54^ were included. Rapid Visual Information Processing^55^ tested sustained attention. Reaction Time^55^ tested reaction time and motor speed. Paired Associates Learning^55^ tested visual memory. Intra-Extra Dimensional Set Shift^55^ tested flexibility or set shift, Stockings of Cambridge^55^ tested planning, and Spatial Working Memory tested spatial working memory.^55^ Two subtests from the Test of Memory and Learning Second Edition, Word Selective Reminding,^55^ and Object Recall,^56^ both tested verbal memory. Six subtests from the Wechsler Intelligence Scale for Children–fourth edition (WISC-IV) were included. Coding^57^ and Symbol Search^57^ both tested speed of processing. Digit Span^57^ and Letter-Number Sequencing^57^ tested verbal working memory. Vocabulary^57^ and Matrices^57^ were used to estimate level of intelligence^58^ (eFigure 1 in Supplement 2).

All raw scores were standardized into *z* scores, and when necessary, direction was reversed to ensure that higher scores indicated better performance. Normal distribution was observed in most cognitive functions, except for visual memory (left skewed), flexibility or set shift (bimodal), and spatial working memory (left skewed).

### Statistical Analysis

Statistical analyses were conducted from February to June 2025. In the main analyses, we estimated the association of high-dose vs standard-dose vitamin D_3_ with the 11 cognitive functions using linear regression. Analyses were performed unadjusted and with multicovariate adjustment for child sex and age at the COPSYCH visit, season of birth, preintervention 25(OH)D levels, and the n-3-LCPUFA intervention (eTable 1 in Supplement 2). Across all analyses, cognitive outcomes were analyzed as standardized *z* scores (mean [SD], 0 [1]), with higher scores indicating better performance.

As a secondary analysis, we estimated the association between preintervention 25(OH)D levels and cognitive functions using crude and adjusted linear regression. Concentrations of 25(OH)D were divided by a factor of 10, so estimates reflected the change per 4-ng/mL increase of 25(OH)D (to convert to nanomoles per liter, multiply by 2.496). A directed acyclic graph guided covariate selection, based on known factors associated with 25(OH)D levels and cognition (eFigure 2 in Supplement 2). In adjusted analyses, we added sex, season of measurement, gestational diabetes, preeclampsia, smoking during pregnancy, alcohol during pregnancy, maternal educational level, and household income as factor variables. Birth weight, gestational age, maternal prepregnancy body mass index, maternal pregnancy inflammation (assessed via interleukin 6 and C-reactive protein), maternal pregnancy diet,^59^ maternal age, paternal age, and age at COPSYCH visit were added as continuous variables.

As sensitivity analyses, we tested the interaction between the vitamin D_3_ intervention and preintervention 25(OH)D levels by adding cross-product terms to the linear regression models. Likewise, we tested the interaction with sex based on known sex differences in neurodevelopment^60^ and the interaction with child 6-month and 6-year 25(OH)D levels based on the hypothesis that higher childhood vitamin D status may enhance the effect of prenatal supplementation. In addition, we tested the n-3-LCPUFA intervention given potential neurodevelopmental interactions.^61^ Second, to explore whether potential supplementation associations were associated with underlying psychopathologic characteristics, we repeated the analyses separately among children with and children without an ADHD or ASD diagnosis. We tested each individual subtest, to identify those linked with observed associations with the cognitive functions. Finally, we conducted an achieved-level analysis defining exposure as maternal postpartum 25(OH)D level of 40 ng/mL or more vs less than 40 ng/mL. Restricted cubic spline regression was used to model associations between postpartum 25(OH)D levels and cognitive outcomes.

All *P* values were from 2-sided tests, and results were deemed statistically significant at *P* < .05. Missing data were not imputed. Analyses were performed using R, version 4.3.1 (R Project for Statistical Computing). The primary RCT results were adjusted using the Benjamini-Hochberg false discovery rate (FDR) correction (5%) applied within cognitive domains (eAppendix and eFigure 3 in Supplement 2).

## Results

### Baseline Characteristics

**Figure 1.**
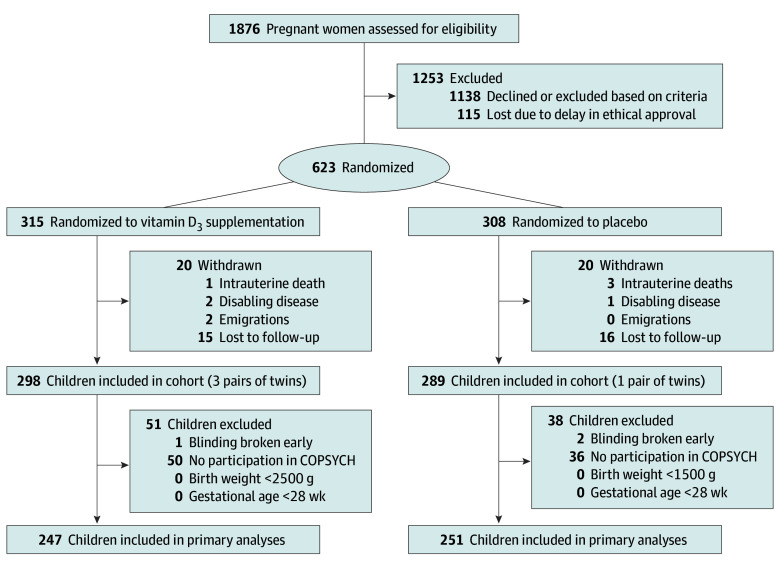
Flow Diagram COPSYCH indicates Copenhagen Prospective Study on Neuro-Psychiatric Development.

Of the 700 mother-child pairs enrolled in the COPSAC_2010_ cohort, 623 were randomized to receive high-dose or standard-dose vitamin D_3_. In total, 498 children (mean [SD] age, 10.3 [0.4] years; 258 boys [51.8%] and 240 girls [48.2%]; 476 White [95.6%] and 22 race or ethnicity other than White [4.4%]) participated in both the COPSYCH visit and the prenatal vitamin D_3_ trial (eTable 2 in Supplement 2). Of these, 251 had been prenatally exposed to placebo and 247 to high-dose vitamin D_3_ (Figure 1).

Extensive baseline characteristics of the included mother-child pairs are outlined in eTable 2 in Supplement 2, and descriptive statistics of cognitive test scores are provided in eTable 3 in Supplement 2. There were no significant differences in preintervention 25(OH)D levels or season of birth between the supplementation and placebo group. Overall median preintervention levels were 30.3 ng/mL (IQR, 23.3-36.9 ng/mL), with 15.0% (74 of 494) having levels lower than 20 ng/mL. Postintervention 25(OH)D levels between the 2 groups were 27.5 ng/mL (IQR, 18.5-36.1 ng/mL) in the placebo group and 41.9 ng/mL (IQR, 34.5-51.0 ng/mL) in the supplementation group. Overall, 36.9% (184 of 498) reached serum 25(OH)D concentrations of 40 ng/mL or more after intervention. This proportion was 57.5% (142 of 247) in the high-dose group and 16.7% (42 of 251) in the standard-dose group (eFigure 4 in Supplement 2).

In the secondary analyses of the week 24 levels, we also included 90 mothers from the cohort who did not participate in the vitamin D_3_ trial. In this combined group, the mean (SD) 25(OH)D levels at pregnancy week 24 were 30.3 (10.1) ng/mL.

### High-Dose Vitamin D_3_ Supplementation and Cognitive Outcomes

The mean (SD) estimated intelligence score for offspring at 10 years was 107.6 (15.0) for the vitamin D_3_ group and 107.8 (13.2) for the placebo group. Of the 11 cognitive functions, vitamin D_3_ supplementation was positively associated with verbal memory (β = 0.17 SD; 95% CI, 0.03-0.32 SD; *P* = .02), visual memory (β = 0.24 SD; 95% CI, 0.06-0.42 SD; *P* = .01), and flexibility or set shift (β = 0.19 SD; 95% CI, 0.01-0.37 SD; *P* = .04) after covariate adjustment (Table 2), but it was no longer associated with flexibility or set shift after multiple test correction (eFigure 5 in Supplement 2). No significant differences were found between the vitamin D_3_ supplementation and placebo groups for the remaining 8 cognitive functions (Table 2).

**Table 2.** Association of High-Dose vs Standard-Dose Vitamin D_3_ Supplementation During Pregnancy With Cognitive Functions at Age 10 Years

Domain	Function	Unadjusted			Adjusted			*q* Value^c^
		No. of children	Estimate (95% CI)^a^	*P* value	No. of children	Estimate (95% CI)^b^	*P* value	
Estimated intelligence	Estimated intelligence	495	−0.01 (−0.19 to 0.16)	.90	491	−0.01 (−0.19 to 0.16)	.89	.89
Processing speed	Speed of processing	498	−0.04 (−0.19 to 0.12)	.65	494	−0.02 (−0.17 to 0.13)	.81	.81
Reaction time	Reaction time	496	0.12 (−0.05 to 0.28)	.17	492	0.11 (−0.05 to 0.28)	.18	.18
Attention	Sustained attention	495	−0.03 (−0.21 to 0.15)	.77	491	−0.03 (−0.21 to 0.16)	.79	.79
Motor function	Motor speed	496	0.10 (−0.07 to 0.27)	.25	492	0.09 (−0.08 to 0.25)	.31	.31
Memory	Verbal memory	497	0.14 (−0.00 to 0.29)	.06	493	0.17 (0.03 to 0.32)	.02	.02
Working memory	Verbal working memory	498	−0.11 (−0.25 to 0.04)	.16	494	−0.01 (−0.25 to 0.04)	.17	.17
Memory	Visual memory	497	0.25 (0.07 to 0.44)	.01	493	0.24 (0.06 to 0.42)	.01	.02
Executive function	Flexibility or set shift	495	0.20 (0.02 to 0.38)	.03	491	0.19 (0.01 to 0.37)	.04	.10
Executive function	Spatial working memory	498	−0.11 (−0.29 to 0.06)	.20	494	−0.12 (−0.30 to 0.06)	.18	.27
Executive function	Planning	497	0 (−0.18 to 0.17)	.96	493	0 (−0.17 to 0.17)	.98	.98

^a^ Estimates represent unadjusted and adjusted mean differences in standardized cognitive outcome scores (*z* scores; mean [SD], 0 [1]) between children prenatally exposed to high-dose vs standard-dose vitamin D_3_ supplementation.

^b^ Adjusted for sex, age at testing, long-chain polyunsaturated fatty acid intervention, season of birth, and preinterventional 25(OH)D level.

^c^ Benjamini-Hochberg false discovery rate (5%) applied across domains. Domains comprising a single function were not subject to multiplicity correction; *q* < .05 considered significant.

### Preintervention 25(OH)D Levels and Cognitive Outcomes

The unadjusted analyses showed no association between preintervention 25(OH)D levels and cognitive functions. After adjustment for a priori chosen potential confounders, only flexibility or set shift showed a positive association (β per 10 nmol/L = 0.05 [95% CI, 0.01-0.09]; *P* = .03); however, this result did not remain significant after FDR correction (Table 3).

**Table 3.** Associations of Maternal 25(OH)D Levels at Week 24 With Offspring Cognitive Functions at Age 10 Years

Domain	Function	Unadjusted			Adjusted			*q* Value^c^
		No. of children	Estimate (95% CI)^a^	*P* value	No. of children	Estimate (95% CI)^b^	*P* value	
Estimated intelligence	Estimated intelligence	584	0 (−0.03 to 0.03)	.93	483	−0.02 (−0.06 to 0.02)	.36	.35
Processing speed	Speed of processing	587	0 (−0.03 to 0.03)	.83	487	0 (−0.03 to 0.04)	.65	.65
Reaction time	Reaction time	585	−0.01 (−0.04 to 0.02)	.54	484	0.01 (−0.03 to 0.04)	.92	.92
Attention	Sustained attention	582	−0.01 (−0.04 to 0.03)	.64	481	0 (−0.04 to 0.04)	.86	.86
Motor function	Motor speed	585	0.01 (−0.02 to 0.04)	.55	484	0.03 (−0.01 to 0.07)	.33	.33
Memory	Verbal memory	587	0.01 (−0.02 to 0.04)	.52	486	−0.01 (−0.04 to 0.03)	.94	.94
Working Memory	Verbal working memory	588	0.02 (−0.01 to 0.05)	.16	487	0 (−0.03 to 0.04)	.70	.70
Memory	Visual memory	586	0.01 (−0.02 to 0.04)	.52	485	0 (−0.04 to 0.04)	.67	.94
Executive function	Flexibility or set shift	584	0.03 (−0.01 to 0.06)	.11	483	0.05 (0.01 to 0.09)	.03	.08
Executive function	Spatial working memory	587	0 (−0.03 to 0.03)	.96	486	0 (−0.04 to 0.04)	.99	.99
Executive function	Planning	586	0 (−0.03 to 0.04)	.79	485	−0.01 (−0.05 to 0.03)	.83	.99

^a^ Estimates represent unadjusted and adjusted regression coefficients for the association between preintervention 25(OH)D levels and standardized cognitive outcomes (*z* scores; mean [SD], 0 [1]). 25(OH)D concentrations were scaled per 4 ng/mL (to convert to nanomoles per liter, multiply by 2.496), such that estimates reflect the change in cognitive performance (SD units) per 4-ng/mL increase in 25(OH)D. Higher scores indicate better cognitive performance.

^b^ Adjusted for sex, birth weight, gestational age, maternal prepregnancy body mass index, season of week 24 measurement, gestational diabetes, preeclampsia, smoking during pregnancy, alcohol during pregnancy, maternal pregnancy interleukin 6 and C-reactive protein levels, maternal educational level, household income, diet, maternal age, paternal age, and child age at Copenhagen Prospective Study on Neuro-Psychiatric Development (COPSYCH) visit.

^c^ Benjamini-Hochberg false discovery rate (5%) applied across domains. Domains comprising a single function were not subject to multiplicity correction; *q* < .05 considered significant.

### Sensitivity Analyses

**Figure 2.**
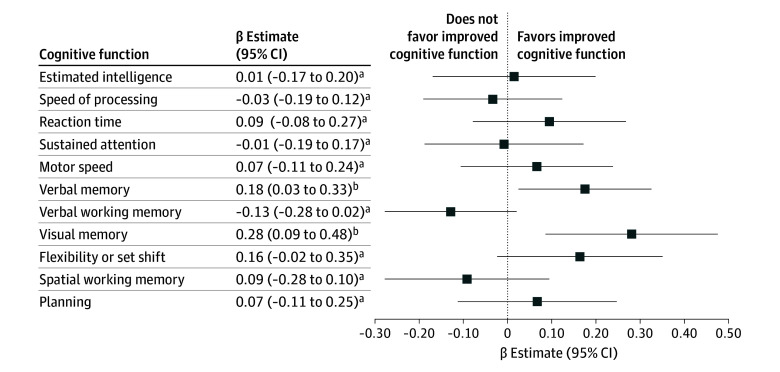
Forest Plot of Association Between High-Dose vs Standard-Dose Vitamin D_3_ Supplementation During Pregnancy and Cognitive Functions at Age 10 Years Among Children Without an Attention-Deficit/Hyperactivity Disorder (ADHD) Diagnosis Analyses were adjusted for child sex, age at assessment, n-3-long-chain polyunsaturated fatty acid supplementation, season of birth, and maternal 25(OH)D concentration at 24 weeks’ gestation. Higher *z* scores indicate better cognitive performance. ^a^*P* ≥ .05. ^b^*P* < .05.

Interaction analyses assessing whether sex, preintervention 25(OH)D, child 25(OH)D levels at 6 months and 6 years, or the n-3-LCPUFA intervention modified the association of the vitamin D_3_ supplementation revealed no significant interactions with verbal memory, visual memory or flexibility or set shift (eTable 4 in Supplement 2). Based on the a priori hypothesis that the association of vitamin D_3_ supplementation with cognitive function would differ by ADHD diagnosis, we examined the associations stratified by ADHD diagnosis (Figure 2). Significant associations were found among children without ADHD in both verbal memory (β = 0.18 SD; 95% CI, 0.03-0.33 SD; *P* = .02) and visual memory (β = 0.28 SD; 95% CI, 0.09-0.48 SD; *P* = .01), but the interaction analysis was not significant (eTable 5 in Supplement 2). Similar patterns were observed when stratifying by ASD; however, these analyses are limited by the low number of children with an ASD diagnosis (n = 12) (eTable 6 in Supplement 2).

Of the 17 individual cognitive tests, vitamin D_3_ supplementation was positively associated in unadjusted analyses with Paired Associates Learning (total errors), part of the memory domain (β = 0.25 SD; 95% CI, 0.07-0.44 SD; *P* = .01), and Intra-Extra Dimensional Set Shift (extra dimensional stage errors) (β = 0.20 SD; 95% CI, 0.02-0.38 SD; *P* = .03), part of executive functioning (eTable 7 in Supplement 2). These remained significant after adjustment, along with Word Selective Reminding (total number recalled) (β = 0.20 SD; 95% CI, 0.03-0.37 SD; *P* = .02), part of the memory domain. The remaining 14 tests showed no significant associations (eTable 7 in Supplement 2), and the association with Intra-Extra Dimensional Set Shift did not remain significant after FDR correction.

Achieved-level analyses comparing maternal postpartum 25(OH)D levels of 40 ng/mL with levels less than 40 ng/mL showed no differences in offspring cognitive outcomes after adjustment for prespecified covariates (eTable 8 in Supplement 2). In addition, no clear threshold was observed in the spline analyses (eFigure 6 in Supplement 2).

## Discussion

In this post hoc analysis of an RCT, we explored the long-term association of high-dose vs standard-dose vitamin D_3_ supplementation during pregnancy with cognitive functions in children at 10 years of age. We found that high-dose supplementation was positively associated with 3 of 11 functions assessed: verbal memory, visual memory, and flexibility or set shift. However, the association with flexibility or set shift was not significant after correction for multiple testing. Observationally, serum 25(OH)D level in pregnancy was associated with only a marginally better flexibility or set shift. Together, these findings support the hypothesis that prenatal vitamin D_3_ exposure may be positively associated with a subset of cognitive functions in childhood.

Although a previous analysis of this RCT reported no significant effect of prenatal vitamin D_3_ supplementation on cognition at 2.5 years using the Bayley scales,^23^ our current findings at 10 years could indicate that the association of vitamin D_3_ supplementation with a subset of cognitive functions may become measurable later in childhood. This finding is supported by literature on cognitive development in children that highlights how cognition, particularly executive functions, becomes increasingly fine-tuned and differentiated throughout childhood.^62,63^

The only other published vitamin D prenatal RCT^49^ reported a positive effect of 2000 IU/d of vitamin D_3_ supplementation from pregnancy week 12 to week 16 until delivery on the language component of the Brigance Screen at 3 to 5 years in offspring, supporting the hypothesis that increasing prenatal vitamin D_3_ supplementation is positively associated with cognitive functioning. Our results suggest a potential association between prenatal vitamin D_3_ supplementation and the memory domain. To our knowledge, no previous human studies have specifically examined the association between gestational vitamin D status and memory among offspring. This finding is supported by 1 existing rat study^64^ reporting a positive association between maternal vitamin D deficiency and impaired memory. A cross-sectional study of adolescents found that higher serum levels of 25(OH)D at 9 to 13 years of age were associated with better performance on visual, but not verbal, memory.^65^

To investigate the potential influence of neurodevelopmental disorders, we tested the association of maternal vitamin D_3_ supplementation with cognitive function among children with or without ADHD. The signal on visual and verbal memory persisted when restricting the analyses to children without the diagnosis, suggesting that the observed signals are not associated with children with ADHD. However, no significant interaction between vitamin D_3_ supplementation and ADHD status was observed. A recent large Danish study reported inverse associations between neonatal 25(OH)D levels and risk of ADHD, ASD, and schizophrenia.^66^ However, a previous study of the vitamin D RCT in the COPSAC_2010_ cohort found no effect of the prenatal supplementation on ADHD diagnosis, although power may have been reduced by the limited number of children with ADHD in the cohort.^24^

Interaction analyses between vitamin D_3_ supplementation and child 6-month and 6-year 25(OH)D levels were nonsignificant, implying that the observed association of supplementation with cognitive functioning is not associated with 25(OH)D levels in early childhood. This finding suggests that prenatal exposure may represent a critical window during which vitamin D may be associated with cognitive development, supporting the idea of a primarily prenatal programming association. However, postnatal levels may be influenced by numerous confounding factors and may not accurately reflect long-term vitamin D status, meaning that small postnatal associations cannot be ruled out.^67^

A previous Danish prospective cohort study showed a positive association between early pregnancy and cord blood levels of 25(OH)D and intelligence among boys at 7 years of age, measured using WISC-V.^31^ In that cohort, the median cord blood level was below 20 ng/mL. In contrast, we observed no association with intelligence in our cohort, where median preintervention levels were more than 30 ng/mL, suggesting that associations with intelligence may be more apparent at lower baseline 25(OH)D levels. Several prospective studies have similarly reported a positive association between vitamin D levels during pregnancy and various cognitive functions in childhood, including intelligence, attention, and executive functions.^25,28,46^ In contrast, other observational studies report no such association, with some focusing specifically on IQ^32,35^ and others assessing specific cognitive functions.^38,41,43^

Because the baseline vitamin D level in this cohort was relatively high, opportunities for neurocognitive benefit may have been biologically constrained. Prior work demonstrated that placental and maternal conversion to 1,25(OH)_2_D is maximized when circulating 25(OH)D approaches 40 ng/mL.^68^ In the present study, we were unable to identify such a threshold effect, likely reflecting limited exposure contrast, as only approximately half of the participants in the high-dose group reached concentrations above this level. Accordingly, the observed effect sizes were modest (SD range, 0.17-0.24), which is expected in a largely vitamin D–sufficient cohort with limited variability in maternal 25(OH)D concentrations and may lead to underestimation of associations in more deficient populations.

A growing body of research indicates that vitamin D supplementation during pregnancy is associated with multiple health benefits among offspring, including improved bone density,^69^ fewer fractures,^69^ and dental health.^70^ Given the cost and timeline required for large-scale trials, the present findings, together with existing evidence, support recommendations for increasing the dose of routine antenatal vitamin D supplementation.

Analyses of observational data revealed only 1 modest association between maternal preintervention serum 25(OH)D levels and flexibility or set shift. Several factors may explain the discrepancy between findings from the supplementation analyses and those based on preintervention levels. Residual confounding may remain despite adjustment for multiple variables. Preintervention levels reflect vitamin D status primarily during the first and second trimesters, whereas supplementation from week 24 is primarily associated with levels during the third trimester. The discrepancy may partly be explained by differences in timing of exposure, as brain regions follow distinct developmental trajectories. The latter half of pregnancy is a period of rapid cortical maturation that may be particularly sensitive to vitamin D.^71,72^

### Strengths and Limitations

Few RCTs have examined the effect of vitamin D_3_ supplementation during pregnancy on offspring cognitive functions.^23,49^ To our knowledge, this is the first RCT to assess cognitive functions among children older than 5 years using performance-based outcomes. The strengths of this study include the large sample size and the deep phenotyping of children enabled by their participation in the COPSAC_2010_ cohort. Furthermore, the cognitive test battery consisted solely of performance-based subtests covering a broad range of cognitive functions. ADHD diagnoses were based on clinical interviews.^51^

This study also has several limitations. It is a post hoc analysis of an RCT that was not prespecified, thus increasing the risk of spurious findings. The high preinterventional mean 25(OH)D level limits our ability to assess potential benefits among participants with low vitamin D status. The large number of cognitive measures included may have limited statistical power to detect significant group differences. The small number of children with ADHD reduces the power to detect subgroup-specific associations. The use of a single neurocognitive assessment at age 10 years may have limited insight into developmental patterns across childhood. Due to the timing of the supplementation, we had the opportunity to examine the potential association of high-dose supplementation only in late pregnancy. In the observational analyses, residual confounding cannot be ruled out. We lack data on parental intelligence and psychopathologic conditions, both of which are heritable.^73,74,75^ However, we do have data on income and educational level, which are known to be associated with intelligence,^76^ and adjusting for these factors did not substantially modify the results. Finally, the generalizability of the study is limited by the predominance of White participants with a high mean 25(OH)D level.

## Conclusions

This post hoc analysis of an RCT suggests that high-dose vitamin D_3_ supplementation from week 24 of pregnancy to 1 week post partum is positively associated with visual memory, verbal memory, and flexibility or set shift among offspring at age 10 years compared with standard-dose vitamin D_3_ supplementation. The associations with memory functions remained significant after FDR correction, while flexibility or set shift did not. Together, these results contribute to the existing evidence on the possible positive cognitive implications of prenatal vitamin D supplementation.

## Notes

Supplement 1.
Trial Protocol and Statistical Analysis Plan

Supplement 2. **eAppendix. **Supplemental Methods
eReferences.
**eTable 1.** Overview of Covariates for the Adjusted Main and Secondary Analyses **eTable 2.** Baseline Characteristics of Participants Included in the RCT and the COPSYCH Project **eTable 3.** Mean Values of all Tests Included in the Cognitive Test Battery From the COPSYCH Visit at Age 10 **eTable 4.** Interaction Analyses of Vitamin D3 Supplementation With Sex, Maternal Week 24 25(OH)D Level, Offspring 6 Months and 6 Year 25(OH)D Levels and N-3-LCPUFA Intervention **eTable 5.** Effect of High-Dose vs Standard-Dose Vitamin D3 Supplementation During Pregnancy on the Cognitive Functions at Age 10, Split by Children With and Without an ADHD Diagnosis, Including P-Interaction **eTable 6.** Effect of High-Dose vs Standard-Dose Vitamin D3 Supplementation During Pregnancy on the Cognitive Functions at Age 10, Split by Children With and Without an ASD Diagnosis, Including P-Interaction **eTable 7.** Effect of High-Dose vs Standard-Dose Vitamin D3 Supplementation on All the Tests Included in the Neurocognitive Test Battery **eTable 8.** Achieved-Level Analysis of Maternal Postpartum 25(OH)D (≥100 vs <100 nmol/L) and Offspring Cognitive Outcomes **eFigure 1.** COPSAC_2010 _and COPSYCH Measures **eFigure 2.** Directed Acyclic Graph: Pre-Interventional 25(OH)D Levels and Childhood Cognition **eFigure 3.** Heatmap: Spearman Correlation Between All Cognitive Tests From the Original COPSYCH Protocol **eFigure 4.** Violin Plot: Change in Maternal 25(OH)D levels From Pre- to Post-Intervention **eFigure 5.** Forest Plot of Effect of Vitamin D3 Supplementation on Cognitive Functions Including q-Values **eFigure 6.** Restricted Cubic Spline Showing the Association Between Maternal Postpartum 25(OH)D Concentration and Cognitive Outcomes

Supplement 3.
Data Sharing Statement
