# Supplementary material for: High-Dose Vitamin D3 Supplementation During Pregnancy and Test-Based Cognitive Performance at Age 10 Years: A Post Hoc Secondary Analysis of a Randomized Clinical Trial
Source: JAMA Netw Open. 2026 May 18;9(5):e2611464. doi: 10.1001/jamanetworkopen.2026.11464 (PMC13184775; doi:10.1001/jamanetworkopen.2026.11464)
Supplement: Supplement 1. — Trial Protocol and Statistical Analysis Plan [file jamanetwopen-e2611464-s001.pdf]

SUPPLEMENT 1 + 2

This supplement contains the following items:

1. Original protocol, final protocol, summary of changes
2. Original statistical analysis plan, final statistical analysis plan, summary of changes

1. Original protocol, final protocol, summary of changes
ORIGINAL PROTOCOL

The following is an English translation of the original protocol in Danish.

Local Ethics Committee: H-B-2009-014; Approved: 23-02-2009
Danish Health and Medicines Authority: 2612-3959; Approved: 23-02-2009
ClinicalTrials.gov: NCT00856947
EudraCT: 2008-007871-26

Aim

To investigate whether supplementation with high-dose vitamin D during third trimester of pregnancy has a favorable effect on the development of asthma and related disorders in the offspring.

Hypothesis

High-dose vitamin D_3 supplementation during third trimester of pregnancy will reduce the risk of developing asthma in the offspring.

Background

Asthma, eczema and allergy are the most common chronic diseases among children and over the past 40 years, the incidence of these diseases has increased in industrialized countries through yet unknown factors in the environment.
Decreased levels of maternal vitamin D in pregnancy and thereby reduced fetal vitamin D levels in utero are among the early environmental exposures suspected to have an influence on the increased incidence of asthma in children.[1] Based on epidemiological studies, a high intake of vitamin D during pregnancy has been associated with protective effects on asthmatic symptoms in young children.[2,3] Preliminary results of a newer study indicates twice the risk of asthmatic symptoms in preschool children with low vitamin D levels at birth compared to children with a high level of vitamin D levels at birth.[4]
The results are consistent with several other studies, which suggest that the population in westernized countries have a reduced supply and level of vitamin D leading to an increased risk of various diseases. E.g., vitamin D levels in the fetus has been associated with the development of schizophrenia, diabetes mellitus and bone development.[5–7] Furthermore, high levels of vitamin D in adults appears to protect against a number of diseases, including bone diseases and cancer. [8–10]
The reason for these reduced levels of vitamin D may be found in the lifestyle of modern society. The majority of our vitamin D supply derives from sun exposure, and because of increasing awareness of harmful effects of sun exposure in relation to skin cancer, our supply of vitamin D has been markedly reduced. This is a recent development, which has led to the hypothesis that the current levels of vitamin D is too low according to the level for which we are genetically programmed.
Vitamin D level is however associated with and highly influenced by other factors as well. Therefore, it is necessary to conduct controlled, blinded studies on the effect of vitamin D supplementation to provide sufficient basis for future recommendations.

Method and trial procedure
The women are recruited from the COPSAC2010 cohort; Local Ethics Committee (H-B-2008-093), Danish Data Protection Agency (2015-41-3696).

The study is a double-blinded, placebo-controlled, randomized parallel group design. 800 pregnant women will be randomized in a 1:1 ratio to intake of either high dose vitamin D supplementation or placebo according to one of the following regimes:

1) Placebo (+ guidance in recommended supplement of vitamin D (400 units daily)) or

2) High dose vitamin D supplement (2400units daily) (+ guidance in recommended supplement of vitamin D (400units daily))

The regimes are administered orally as 2 tablets daily.

Blinding and randomization are carried out by the Capital Region Pharmacy and stratified according to treatment group in the fish oil intervention study (ClinicalTrials.gov: NCT00798226). This allows for equal numbers receiving high dose vitamin D supplementation in both the fish oil active group and the fish oil placebo group.

The intervention is initiated at the beginning of the third trimester (pregnancy week 24) and continued until 1st visit to the COPSAC clinic after birth at week 1-2 postpartum. At the clinical visit in pregnancy week 24, the women will be provided with the intervention treatment and interviewed about current daily vitamin D intake and history of diseases likely to influence vitamin D levels. At pregnancy week 36 adherence to the regime will be assessed by interview at the COPSAC clinic. Furthermore, the women will be instructed to return the remaining tablets at the end of the intervention for evaluation of their compliance.

At pregnancy week 24 and 1st visit after birth a blood sample will be drawn from the mother in order to measure 25-OH-vitamin D, total calcium, parathyroid-hormone (PTH) and alkaline phosphatase.

Inclusion criteria
The study population consists of healthy pregnant women and their children participating in the COPSAC2010 cohort. Vitamin D supplements are administered during the third pregnancy trimester. The women will be included in the study independent of residence, age, race and social status during week 22-26 of pregnancy.

Exclusion criteria
Pregnant women are excluded from the trial, if they carry a disease leading to an increased risk of potential side effects from high-dose vitamin D supplementation: Endocrinologic disease in the form of calcium metabolic disorders, parathyoroidea disease, thyroid disorders or type 1 diabetes; Tuberculosis; Sarcoidosis or illness requiring chronic treatment with diuretics or heart medications, including calcium channel blockers or if they have a current intake of vitamin D supplements over the recommended dose.

Risks and disadvantages:
Known potential adverse effects of vitamin D intoxication is hypercalcemia and accompanying symptoms such as loss of appetite, nausea, vomiting, weight loss, headache, lethargy, fatigue, confusion and renal impairment. These side effects are not found by the administration of vitamin D

in physiological doses. Vitamin D intoxication occurs only by the intake of very high doses of Vitamin D (4 times higher doses than administered in our study). In order to avoid administering vitamin D supplements to women with a high initial level, women with an intake above the recommended dose in the previous 6 months are excluded. Expected disadvantages related to blood sample procedures and are temporary in nature without the risk of permanent injury.

Ethical aspects

Oral vitamin D supplement has been shown to be safe and non-toxic in many randomized trials, including studies involving pregnant women. The risk of adverse effects in the pregnant woman or the fetus is suspected to be minimal. Based on the previous studies, it is expected that a large proportion of the participating women will have a daily low Vitamin D level, and therby vitamin D supplementation to these women will be a health benefit. The control group receive recommended dose of vitamin D, and ethical problems in relation to sufficient treatment of the control group is thereby not a problem.

We believe that the study as outlined above is ethically acceptable and randomized trials of vitamin D supplements are necessary for future recommendations of vitamin D intake.

FINAL PROTOCOL
Unique Protocol ID: 2008-007871-26
NCT00856947

Official Title: Vitamin D Supplementation During Pregnancy for Prevention of Asthma in Childhood: An Interventional Trial in the ABC (Asthma Begins in Childhood) Cohort

Study Start: March 2009

Human Subjects Review: Board Status: Approved Approval Number: 2612-3959

Study Description

Brief Summary:
The aim of this study is to prevent asthma symptoms (recurrent wheeze) in childhood by supplementation with high dose vitamin D to the mother during pregnancy. Participants are mothers and children of the ABC (Asthma Begins in Childhood) cohort. Mothers are recruited during pregnancy and receive daily supplement with 2400 IU of Vitamin D3 or placebo from week 24 of gestation to 1 week after delivery. In addition all mothers are advised to take the recommended dose of 400 IU vitamin D daily. The mothers in ABC also participate in an interventional trial with fish oil supplementation, and the vitamin D randomization is stratified by fish oil treatment group. The child is followed with acute and planned vits at the research unit, and wheeze is diagnosed according to predefined algorithms.

Arms and interventions:
Active Comparator: Vitamin D
Dietary supplement: 2400 IU Vitamin D_3 (2 tablets of 1200 IU) from week 24 of gestation to 1 week after delivery.
Dietary Supplement: Cholecalciferol D_3 2 tablets of 1200 IU daily from week 24 of gestation to 1 week after delivery. Vitamin D from Camette, Denmark

Placebo Comparator: Placebo
Placebo: 2 placebo tablets with no active substance, identical to the active tablets, from week 24 of gestation to 1 week after delivery
Placebo tablet: 2 tablets containing no active substance. Placebo tablets from Camette, Denmark

Outcome Measures
Primary Outcome Measure:
1. Persistent wheeze
Age at onset of persistent wheeze diagnosed according to predefined algorithm of recurrent troublesome lung
symptoms, response to treatment and relapse after withdrawal of treatment
[Time Frame: 0 to 3 years of age]

Secondary Outcome Measure:
2. Infections
Main analysis:
• Number of lower respiratory tract infections registered in daily diaries

Secondary analyses:
• Acute otitis media
• Number of upper respiratory tract infections
• Number of other infections
• Total number of infections
[Time Frame: 0 to 3 years of age]

3. Allergic sensitization
Allergic sensitization at 6 and/or 18 months assessed by skin prick test and specific IgE in blood
[Time Frame: 6 and 18 months of age]

4. Eczema
Age at onset of eczema diagnosed prospectively by research doctors according to predefined algorithm based upon
Hanifin and Rajka criteria
[Time Frame: 0 to 3 years of age]

5. Mothers levels of 25-OH-Vitamin D, PTH, Calcium, alkaline phosphatase
[Time Frame: 1 week after delivery]

6. Growth
[Time Frame: 0 to 3 years of age]

7. Asthma exacerbations
Age at onset of severe asthma exacerbation diagnosed by predefined criteria of acute severe asthma requiring oral/
high dose inhaled steroids or acute hospital contact
[Time Frame: 0 to 3 years of age]

8. Neurological development
Main analysis:
• Cognitive development assessed at 2½ years using the cognitive part of Bayley Scales of Infant and Toddler development, third edition

Secondary analyses:
• Milestone development monitored prospectively by the parents using a registration form based on The Denver Development Index and WHO milestones registration (combined assessment by principal component analysis)
• Language development assessed at 1 and 2 years of age with the Danish version of The MacArthur Bates Communicative Developmental Inventory (CDI)
• The child´s general development (language, fine and gross motor, social and problem solving) at 3 years of age assessed with Ages and stages Questioner, third edition (ASQ-3)
[Time Frame: 0-3 years]

9. Growth
Main analysis:
• Body composition (fat mass and bone mineral density) assessed by DEXA scan at 3 years of age
Secondary analysis

• Development of BMI from birth to 3 years assesses longitudinally in the research clinic
[Time Frame: 0-3 years]

10. Systemic immune status
Main analysis Immune status at 18 months measured in stimulated whole blood as cytokine release (combined assessments by prinicipal component analyses)
Secondary analyses Composition of immune cell subsets in whole blood at birth and at 18 months of age
[Time Frame: 18 months]

11. Airway mucosal immune status
Immune status measured in airway mucosal lining fluid at 4 weeks and 2 years of age (combined assessments by
prinicipal component analyses for each age point)
[Time Frame: 4 weeks and 2 years]

12. 17q21 genotype and sphingolipid metabolites
In a secondary analyses, we will determine the effect of 17q21 genotype on the efficacy of vitamin D supplementation in the prevention of asthma/wheeze. We will compute hazard ratios for the reduction in asthma/wheeze risk associated with prenatal supplementation, stratified by rs12936231. rs12936231 is a functional SNP influencing expression of ORMDL3, and given the role of ORMDL3 as a key sphingolipid biosynthesis regulator, we will subsequently investigate the relative abundance of sphingolipids between those in the vitamin D Intervention arm and those in the placebo group, stratified by 17q21 genotype. Finally we will identify interactions between prenatal vitamin D supplementation, rs12936231 genotype and sphingolipid metabolism in the risk of asthma/wheeze by age three.
[Time Frame: 6 months]

13. Dental health
Caries and enamel defects (molar incisor hypomineralization, MIH) determined at a dental examination at age 6 years.
[Time Frame: 6 year]

Other Pre-specified Outcome Measures:
14. Asthma
Asthma diagnosed from age 3 to 10 years based on the same predefined algorithm of recurrent troublesome lung symptoms, response to treatment after withdrawal of treatment, which was used for persistent wheeze at age 0-3 in phase 1 of the study. Primary outcome in phase 2 is current asthma at specific visits till age 10 years, which is diagnosed in children fulfilling the persistent wheeze algorithm at any point during the first 10 years of life and still needing inhaled corticosteroids at specific visits (3, 4, 5, 6, 8 and 10 years of age) to control the symptoms.

Asthma exacerbations:
Age at onset and number of severe asthma exacerbation diagnosed by predefined criteria of acute severe asthma requiring oral/ high dose inhaled steroids or acute hospital contact
[Time Frame: 3-10 years of age]

15. Lung function measurements

Spirometry measuring airflow assessed by FEV1, MMEF and FEV1/FVC ratio at age 5, 6, 8 and 10 years and airway resistance (sRaw) measured by plethysmography at age 3, 4, 5, 6, 8 and 10 years.
Multiple breath wash-out using SF6 and N2 as inert gasses to determined LCI, Scond and Sacin at ages 3, 4 and 5 years.
Bronchial reactivity: [Time Frame: at 6 years of age] Provocative dose of methacholine leading to a 20% drop in FEV1 from baseline (PD20 value) at age 6 years.
Airway inflammation: [Time Frame: 6-10 years of age] Measurement of fractional exhaled nitric oxide (FeNO) at age 6, 8 and 10 years
[Time Frame: 3-10 years of age]

16. Infections
Prescribed medicine for infections. Types and length of infections
[Time Frame: 3-10 years of age]

17. Growth
Anthropometrics:
Clinical follow up on the development of weight in kg (calibrated digital weight scales), height in cm (Harpenden stadiometer), waist-, thorax- and head circumference in cm (using tape; 3 times each) at every visit till age 10 years assessed longitudinally in the research clinic.

Body composition:
Body composition measured as fat mass, lean mass, bone mineral content (BMC) and bone mineral density assessed (BMD) by DXA scans at 6 years of age.
Body impedance measurements at 10 years of age.
[Time Frame: 3- 10 years of age]

18. Cognitive function
The following cognitive functions will be covered by paper-and-pencil-tests:
• The verbal memory.
• The Vocabulary and Matrices subtests from (WISC-IV) to estimate intelligence.
• Verbal working memory.
• Processing speed.
• Mental flexibility..
• Fine motor dexterity will be assessed with the Grooved Pegboard Test

The following cognitive functions will be assessed using the Cambridge Neuropsychological Test Automated Battery subtests:
• Sensorimotor functioning.
• Mental response speed and motor speed will be assessed with the Reaction Time task;
• Working memory capacity.
• Working memory and strategy formation.
• Attentional set formation, maintenance, shifting, and flexibility of attention.
• Response inhibition.
• Visual learning and memory.
• Sustained attention.
• Facial affect recognition.
[Time Frame: 10 years of age]

19. Behavioral and psychopathological dimensions.
Questionnaires will be administered to the parents. ADHD-RS questionnaire at 8 and 10 years of age. Strength and Difficulties Questionnaire (SDQ) at 6, 8 and 10 years of age. Social Responsive Scale, Second version (SRS-2) at 10 years of age. The Behavior Rating Inventory of Executive Function 2nd edition (BRIEF-2) at 10 years of age. The Child Behavior Checklist school-age version (CBCL) at 10 years of age. Behavioral and psychopathological dimensions will be assessed at 10 years of age with the semi-structured clinical interview Schedule for Affective Disorders and Schizophrenia for School-Age Children - Present and Lifetime Version (K-SADS-PL) first with a parent and next with the child.
The child assessor will rate potential behavioral and emotional problems observed during the cognitive test session using the Test Observation Form (TOF).
The Magical Thinking Questionnaire (MTQ) will be given to the child.
[Time Frame: 6- 10 years of age]

20. MRI scanning of the Brain
Following technics will be used. Structural MRI scanning to distinguish between grey and white matter. Diffusion weighted imaging to register the fiber directions in the brain. Magnetisation transfer imaging to measure the magnetisation transfer ratio reflecting the interaction between macromolecular protons and the free water protons of tissue, and this technique in addition to quantitative T1-mapping will be used to assess myelination in the developing brain.
Magnetic resonance spectroscopy (MRS) allows for non-invasive measurement of metabolites.
Phase-contrast MR angiography to detect the total cerebral blood flow. zsArterial spin labelling to measure cerebral blood perfusion.
[Time Frame: 10 years of age]

21. Eczema
Age at onset of eczema diagnosed prospectively by research doctors according to predefined algorithm based upon Hanifin and Rajka criteria and severity determined by SCORAD score. In phase 2 current eczema at specific visits till age 10 years will be evaluated.
[Time Frame: 3-10 years of age]

22. Allergic sensitization/atopy
Allergic sensitization at 6 and 10 years of age assessed by skin prick test (ALK-Abelló, Denmark) and specific IgE in blood (ImmunoCAP, PHarmacia Diagnostics AB, Sweden) and total-IgE level and blood eosinophil count measured at the same timepoints.
[Time Frame: 3-10 years of age]

23. Airway mucosal immune status
Immune status measured in airway mucosal lining fluid at 3, 6 and 10 years of age (combined assessments by principal component analyses for each age point).
[Time Frame: 3-10 years of age]

24. allergic rhinitis
Allergic sensitization combined with symptom recording of troublesome congestion or sneezing or runny nose upon relevant exposure to allergens at age 3, 4, 5, 6, 8 and 10 years.
[Time Frame: 3-10 years of age]

Overview of clinical tests; phase 2:

**Children**	**4 years**	**5 years**	**6 years**	**Investigations of biologic material**
Airway and eczema diary	x	x	x	
Doctor examination			x	
Tympanometry			x	
Blood pressure			x	
Growth details	x	x	x	
Nose filter			x	Immune system
Electronic nose measure			x	Exhaled air
Urine sample			x	Interleukins, leukotrienes and metabolic products
Blood sample			x	Immune system, epigenetics and metabolic product
Skin prick test			x	Allergy
Hair sample			x	Cotinine (nicotine product)
Faeces sample	x	x	x	Microbiological colonization
Skin swab			x	Microbiological colonization
Throat swab			x	Microbiological colonization
Nasal scrape*			x	mRNA - gene expression
Activity measure			x	
DXA scan			x	Lean mass, fat mass and bone mineral content/ density
Spirometry			x	
Bodybox	x		x	
Multiple breath washout	x		x	
Metacholine provocation*			x	
FeNO measure*			x	
Child Behavior Checklist*			x	Mentality and behavior
**Parents**				
Interview - child environment	x	x	x	
Interview - child asthma and allergy	x	x	x	

**New tests*

SUMMARY OF CHANGES

Changes to the original protocol are indicated in https://clinicaltrials.gov/ct2/show/NCT00856947.

Briefly, these encompass introduction of novel assessments, including evaluation of cognition and psychopathology, growth, systemic immune status and airway mucosal immune status.

Reference List

1 Litonjua AA, Weiss **ST**. Is vitamin D deficiency to blame for the asthma epidemic? *J Allergy Clin Immunol* 2007;**120**:1031–5. doi:10.1016/j.jaci.2007.08.028

2 Devereux G, Litonjua AA, Turner SW, *et al.* Maternal vitamin D intake during pregnancy and early childhood wheezing. *Am J Clin Nutr* 2007;**85**:853–9.

3 Camargo CA, Rifas-Shiman SL, Litonjua AA, *et al.* Maternal intake of vitamin D during pregnancy and risk of recurrent wheeze in children at 3 y of age. *Am J Clin Nutr* 2007;**85**:788–95.

4 Camargo CA, Ingham T, Wickens K, *et al.* Cord-Blood 25-Hydroxyvitamin D Levels and Risk of Respiratory Infection, Wheezing, and Asthma. *Pediatrics* 2011;**127**:e180–7. doi:10.1542/peds.2010-0442

5 McGrath J, Selten J-P, Chant D. Long-term trends in sunshine duration and its association with schizophrenia birth rates and age at first registration — data from Australia and the Netherlands. *Schizophr Res* 2002;**54**:199–212. doi:10.1016/S0920-9964(01)00259-6

6 Hyppönen E, Läärä E, Reunanen A, *et al.* Intake of vitamin D and risk of type 1 diabetes: a birth-cohort study. *The Lancet* 2001;**358**:1500–3. doi:10.1016/S0140-6736(01)06580-1

7 Javaid M, Crozier S, Harvey N, *et al.* Maternal vitamin D status during pregnancy and childhood bone mass at age 9 years: a longitudinal study. *The Lancet* 2006;**367**:36–43. doi:10.1016/S0140-6736(06)67922-1

8 Bischoff-Ferrari HA, Giovannucci E, Willett WC, *et al.* Estimation of optimal serum concentrations of 25-hydroxyvitamin D for multiple health outcomes. *Am J Clin Nutr* 2006;**84**:18–28.

9 Holick MF. Vitamin D deficiency. *N Engl J Med* 2007;**357**:266–81. doi:10.1056/NEJMra070553

10 Adams JS, Clemens TL, Parrish JA, *et al.* Vitamin-D Synthesis and Metabolism after Ultraviolet Irradiation of Normal and Vitamin-D-Deficient Subjects. *N Engl J Med* 1982;**306**:722–5. doi:10.1056/NEJM198203253061206

2. Original statistical analysis plan, final statistical analysis plan, summary of changes

ORIGINAL STATISTICAL ANALYSIS PLAN

Outcome definitions:

Primary outcome
Persistent wheeze: Age at onset of persistent wheeze diagnosed according to a predefined algorithm of recurrent troublesome lung symptoms, response to treatment and relapse after withdrawal of treatment

Secondary outcomes
Asthma exacerbations: Age at onset of severe asthma exacerbations diagnosed by predefined criteria of acute severe asthma requiring oral/high dose inhaled steroids or acute hospital contact
Eczema: Age at onset of eczema diagnosed prospectively by research doctors according to predefined algorithm based upon Hanifin and Rajka criteria

Allergic sensitization: Allergic sensitization at 6 and/or 18 months of age assessed by skin prick test and specific IgE in blood.

Infections: Main analysis: Number of lower respiratory tract infections registered in daily diaries
Secondary analyses: Acute otitis media, number of upper respiratory tract infections, number of other infections, total number of infections

Mothers levels of 25-OH-Vitamin D, PTH, Calcium, alkaline phosphatase

Growth: Anthropometric measurements in the clinic.
Body composition (fat mass and bone mineral density) assessed by DXA scan at 3 years of age
Development of BMI from birth to 3 years assesses longitudinally in the research clinic

Statistical analyses:

The effect of high-dose Vitamin D_3 supplementation on age at onset of persistent wheeze, lower respiratory infections, and eczema is analyzed by Cox proportional hazards regression, where p-values correspond to Wald tests. The children are retained in the model from birth until age of diagnosis, drop out, or age at their last clinic visit before the RCT was unblinded.
The effect of Vitamin D_3 supplementation on the cross-sectional end-points asthma and allergic sensitization is analyzed by logistic regression, whereas the effect on number of wheezy episodes and upper respiratory infections is analyzed by a generalized estimating equation (GEE) Poisson regression model.
The effect on airway immunology is analyzed by calculating geometric mean ratios of each mediator in the high-dose Vitamin D_3 vs. control group and by a principal component analysis (PCA) capturing the overall immunological trends in the data and their relation to the intervention analyzed by Wilcoxon rank sum test. Initially, the mediator levels were log-transformed. Prior to the PCA the variables ware scaled to unit variance.
The primary analysis of persistent wheeze is presented crude and adjusted for sex, birth season, maternal Vitamin D level at randomization, and the n-3 LCPUFA RCT.

A significance level of 0.05 is used in all types of analyses.

Additional secondary endpoints:
The novel assessments introduced in the cohort resulted in additional secondary end-points:

Airway mucosal immune status
Description: Immune status measured in airway mucosal lining fluid at 4 weeks and 2 years of age (combined assessments by prinicipal component analyses for each age point)

Systemic immune status
Description:
Main analysis: Immune status at 18 months measured in stimulated whole blood as cytokine release (combined assessments by principal component analyses)
Secondary analysis: Composition of immune cell subsets in whole blood at birth and at 18 months of age
Neurological development 0-3 years
Description:
Main analysis: Cognitive development assessed at 2½ years using the cognitive part of Bayley Scales of Infant and Toddler Development, third edition
Secondary analyses: 1) Milestone development monitored prospectively by the parents using a registration form based on The Denver Development Index and WHO milestones registration (combined assessment by principal component analysis); 2) Language development assessed at 1 and 2 years of age with the Danish version of The MacArthur Bates Communicative Developmental Inventory (CDI); 3) The child´s general development (language, fine and gross motor, social and problem solving) at 3 years of age assessed with Ages and stages Questioner, third edition (ASQ-3)

Growth:
Body composition: Body composition measured as fat mass, lean mass, bone mineral content (BMC) and bone mineral density assessed (BMD) through DXA scans at 6 years of age.
Anthropometrics: Clinical follow up on the development of weight in kg (calibrated digital weight scales), height in cm (Harpenden stadiometer), waist-, thorax- and head circumference in cm (using tape; 3 times each) at every visit till age 10 years assessed longitudinally in the research clinic.

FINAL STATISTICAL ANALYSIS PLAN

Statistics

The effect of high-dose vs standard-dose vitamin D3 on the eleven cognitive functions were analyzed by linear regression analyses. We tested for interaction between the vitamin D3 intervention and pre-interventional levels of 25(OH)D, sex, child 6-month 25(OH)D levels, child 6-year 25(OH)D levels and ADHD diagnosis by adding cross-products to the models. The main analyses were repeated separately among children with and without an ADHD diagnosis.
All main analyses were performed crude and adjusted for child sex and age at the COPSYCH visit, season of birth, pre-intervention 25(OH)D levels, and the n-3 LCPUFA intervention.

Secondary analyses estimated the association between pre-intervention 25(OH)D levels and cognitive functions by linear regression in crude and adjusted analyses. 25(OH)D concentrations were divided by a factor of 10, so effect estimates reflect the change in outcome per 10 nmol/L increase of 25(OH)D.

The effect of high-dose vs standard-dose vitamin D3 was tested on each individual subtest using linear regression analyses.
Statistical significance was set as <0.05. Analyses were performed using R statistical software version 4.3.1.
Primary results of the RCT were corrected for multiple testing using FDR at 5%.

SUMMARY OF CHANGES

Specification of the statistical approach regarding the effect of vitamin D3 on cognitive abilities.
