# Supplementary material for: High-Dose Vitamin D3 Supplementation During Pregnancy and Test-Based Cognitive Performance at Age 10 Years: A Post Hoc Secondary Analysis of a Randomized Clinical Trial
Source: JAMA Netw Open. 2026 May 18;9(5):e2611464. doi: 10.1001/jamanetworkopen.2026.11464 (PMC13184775; doi:10.1001/jamanetworkopen.2026.11464)
Supplement: Supplement 2. — eAppendix. Supplemental Methods eReferences. eTable 1. Overview of Covariates for the Adjusted Main and Secondary Analyses eTable 2. Baseline Characteristics of Participants Included in the RCT and the COPSYCH Project eTable 3. Mean Values of all Tests Included in the Cognitive Test Battery From the COPSYCH Visit at Age 10 eTable 4. Interaction Analyses of Vitamin D3 Supplementation With Sex, Maternal Week 24 25(OH)D Level, Offspring 6 Months and 6 Year 25(OH)D Levels and N-3-LCPUFA Intervention eTable 5. Effect of High-Dose vs Standard-Dose Vitamin D3 Supplementation During Pregnancy on the Cognitive Functions at Age 10, Split by Children With and Without an ADHD Diagnosis, Including P-Interaction eTable 6. Effect of High-Dose vs Standard-Dose Vitamin D3 Supplementation During Pregnancy on the Cognitive Functions at Age 10, Split by Children With and Without an ASD Diagnosis, Including P-Interaction eTable 7. Effect of High-Dose vs Standard-Dose Vitamin D3 Supplementation on All the Tests Included in the Neurocognitive Test Battery eTable 8. Achieved-Level Analysis of Maternal Postpartum 25(OH)D (≥100 vs <100 nmol/L) and Offspring Cognitive Outcomes eFigure 1. COPSAC2010 and COPSYCH Measures eFigure 2. Directed Acyclic Graph: Pre-Interventional 25(OH)D Levels and Childhood Cognition eFigure 3. Heatmap: Spearman Correlation Between All Cognitive Tests From the Original COPSYCH Protocol eFigure 4. Violin Plot: Change in Maternal 25(OH)D levels From Pre- to Post-Intervention eFigure 5. Forest Plot of Effect of Vitamin D3 Supplementation on Cognitive Functions Including q-Values eFigure 6. Restricted Cubic Spline Showing the Association Between Maternal Postpartum 25(OH)D Concentration and Cognitive Outcomes [file jamanetwopen-e2611464-s002.pdf]

# Supplementary Online Content

Frederiksen OF, Jepsen JRM, Brustad N, et al. High-dose vitamin $D_3$ supplementation during pregnancy and test-based cognitive performance at age 10 years: a post hoc secondary analysis of a randomized clinical trial. *JAMA Netw Open.* 2026;9(5):e2611464. doi:10.1001/jamanetworkopen.2026.11464

**eAppendix.** Supplemental Methods
**eReferences.**
**eTable 1.** Overview of Covariates for the Adjusted Main and Secondary Analyses
**eTable 2.** Baseline Characteristics of Participants Included in the RCT and the COPSYCH Project
**eTable 3.** Mean Values of all Tests Included in the Cognitive Test Battery From the COPSYCH Visit at Age 10
**eTable 4.** Interaction Analyses of Vitamin D3 Supplementation With Sex, Maternal Week 24 25(OH)D Level, Offspring 6 Months and 6 Year 25(OH)D Levels and N-3-LCPUFA Intervention
**eTable 5.** Effect of High-Dose vs Standard-Dose Vitamin D3 Supplementation During Pregnancy on the Cognitive Functions at Age 10, Split by Children With and Without an ADHD Diagnosis, Including P-Interaction
**eTable 6.** Effect of High-Dose vs Standard-Dose Vitamin D3 Supplementation During Pregnancy on the Cognitive Functions at Age 10, Split by Children With and Without an ASD Diagnosis, Including P-Interaction
**eTable 7.** Effect of High-Dose vs Standard-Dose Vitamin D3 Supplementation on All the Tests Included in the Neurocognitive Test Battery
**eTable 8.** Achieved-Level Analysis of Maternal Postpartum 25(OH)D (≥100 vs <100 nmol/L) and Offspring Cognitive Outcomes
**eFigure 1.** $COPSAC_{2010}$ and COPSYCH Measures
**eFigure 2.** Directed Acyclic Graph: Pre-Interventional 25(OH)D Levels and Childhood Cognition
**eFigure 3.** Heatmap: Spearman Correlation Between All Cognitive Tests From the Original COPSYCH Protocol
**eFigure 4.** Violin Plot: Change in Maternal 25(OH)D levels From Pre- to Post-Intervention
**eFigure 5.** Forest Plot of Effect of Vitamin D3 Supplementation on Cognitive Functions Including q-Values
**eFigure 6.** Restricted Cubic Spline Showing the Association Between Maternal Postpartum 25(OH)D Concentration and Cognitive Outcomes

This supplementary material has been provided by the authors to give readers additional information about their work.

© 2026 Frederiksen OF et al. *JAMA Network Open.*

## **eAppendix.** Supplemental Methods

**Study intervention**
The randomization was performed at the Pharmacy of Glostrup by a computer-generated list of random numbers. The randomization was performed by an external investigator with no additional involvement in the RCT.

**Serum measures of 25(OH)D**
One serum 25(OH)D measure of 0.0 was corrected to half of the lowest observed value of 25(OH)D.

The obtainment of serum vitamin D measures has been described previously in Sass et al[1]: *"The venous blood samples obtained before and after the intervention were centrifuged for 10min at 4300rpm to separate serum, and thereafter frozen at -80oC until analysis. The serum samples were transported on dry ice for duplicate analyses for 25-hydroxyvitamin D2(25(OH)-Vitamin D2) and 25(OH)-Vitamin D3 at the Dept. of Clinical Biochemistry, Aarhus University Hospital, Denmark. Serum 25-hydroxyvitamin D levels were analysed by isotope dilution liquid chromatography-tandem mass spectrometry (LC-MS/MS)[2,3]. Calibrators traceable to NIST SRM 972 (Chromsystems, DE) were used."*

Serum 25-hydroxyvitamin D (25(OH)D) concentrations were measured and primarily reported in nmol/L. In sections where comparison with international literature required alternative units, values were additionally expressed in ng/mL

The following standard conversion factor was applied: 1 ng/mL = 2.496 nmol/L

Accordingly, concentrations in nmol/L were converted to ng/mL by dividing by 2.496. Unless otherwise specified, all values are presented in nmol/L.

**Outcome measures**
Details on inter-rater reliability of agreement is published here: https://doi.org/10.1016/j.psychres.2023.115481

The cognitive domains described in the COPSYCH protocol were refined post hoc to reduce the number of outcomes. When possible, we chose to prioritize the automated Cambridge Neuropsychological Test Battery (CANTAB) tests over paper-and-pencil tests in cases where both tests assessed the same cognitive function. We also prioritized Wechsler Intelligence Scale for Children - fourth edition (WISC-IV) measures as these are well-validated and widely used in previous literature[4–6].

Pearson correlation analyses were performed for all functions that were indexed by more than one test outcome to determine whether test scores could be aggregated into a single composite score (e*Figure 3)*. An acceptable correlation threshold was set to ± 0.4. When two tests did not meet the threshold, one was excluded. This applied to only the spatial working memory function that was indexed by Spatial Working Memory task (SWM) and Spatial Span task (SS). We included SWM rather than SS as literature has shown it is a more effective measure of executive function[7].
The estimated General Ability Index (GAI) from the WISC-IV estimated overall intelligence based on verbal comprehension (Vocabulary) and perceptual reasoning (Matrices) indices. Each subtest outcome score was age-corrected and multiplied by three, acting as a proxy for the absent tests in its respective category, and their sum was converted to GAI score using the General Ability Index Conversion Table with Danish Norms[8].

In total, seven computer-based subtests were included from CANTAB[9]. Rapid Visual Information Processing[10] assessed sustained attention with a working memory component. The child had to watch a stream of moving numbers and respond whenever a specific three-number sequence appeared. Reaction Time[10] consisted of a screen with one (simple mode) or five circles (five-choice mode). The child had to select a circle when it changed color, movement time was a measure of motor speed, while time to react (i.e. time to lift finger from screen) assessed reaction time. Paired Associates Learning[10] assessed visual memory. Boxes on the screen were automatically opened and one or more contained a pattern, after closing the child chose the box where a specific pattern was located. The outcome was adjusted to include scores for all potential rounds, allowing comparison regardless of how many rounds the child completed.

The following CANTAB tests assessed executive functioning; Intra-Extra Dimensional Set Shift[10] measured the ability to shift attention by requiring the child to choose between patterns, learning which is correct through feedback. In the process rule changes shift to a new pattern (extra-dimensional), where the outcome was extra-

© 2026 Frederiksen OF et al. *JAMA Network Open.*

dimensional stage errors. Spatial Working Memory[10] required the child to find hidden tokens in boxes, while avoiding previously searched boxes, as each box held only one token. Stockings of Cambridge[10] measured planning by having the child recreate a pattern of balls by moving one at a time and using as few moves as possible. For detailed information on all the included tests from CANTAB see official website: https://cambridgecognition.com/

The Word Selective Reminding and Object Recall subtests from Test of Memory and Learning (TOMAL-2) both assessed verbal memory. For Word Selective Reminding[11] the examiner read 12 words aloud which the child was asked to recall. If they forgot one or more words, the examiner provided reminders. The child completed six trials. In Object Recall[11], pictures of objects were shown and there were no reminders. The child completed five trials.

Six subtests from WISC-IV were included. Coding[12] and Symbol Search[12] both measured speed of processing. Digit Span[12] and Letter-Number Sequencing[12] assessed verbal working memory. Vocabulary[12] and Matrices[12] estimated intelligence.

**Statistical methods**
Statistical variables were expressed as mean and standard deviation (SD) for normally distributed variables or median and interquartile range (IQR) for skewed data. Categorical variables were reported as numbers and percentages.

The main analyses were adjusted for sex and age at COPSYCH visit as the scores from the cognitive test battery are raw and unadjusted.
For the secondary analyses we constructed a directed acyclic graph to determine appropriate covariates a priori, based on known influencers of 25(OH)D levels and cognition[13–20] (*eFigure 1)*.

The main RCT analyses were adjusted using the Benjamini-Hochberg FDR correction (5%). The analyses were corrected within the cognitive domains. Intercorrelations within the domains are shown in eFigure 3. Domains comprising of a single cognitive function were therefore not subject to multiplicity correction. Specifically, FDR adjustment was applied to the Memory domain (verbal memory and visual memory, 2 comparisons) and the Executive Function domain (flexibility/set shift, spatial working memory and planning, 3 comparisons).

© 2026 Frederiksen OF et al. *JAMA Network Open.*

## eReferences.


1.Sass L, Vinding RK, Stokholm J, et al. High-Dose Vitamin D Supplementation in Pregnancy and Neurodevelopment in Childhood: A Prespecified Secondary Analysis of a Randomized Clinical Trial. JAMA Netw Open. 2020;3(12):e2026018. doi:10.1001/jamanetworkopen.2020.26018 1

2.Højskov CS, Heickendorff L, Møller HJ. High-throughput liquid-liquid extraction and LCMSMS assay for determination of circulating 25(OH) vitamin D3 and D2 in the routine clinical laboratory. Clin Chim Acta Int J Clin Chem. 2010;411(1-2):114-116. doi:10.1016/j.cca.2009.10.010

3.Maunsell Z, Wright DJ, Rainbow SJ. Routine isotope-dilution liquid chromatography-tandem mass spectrometry assay for simultaneous measurement of the 25-hydroxy metabolites of vitamins D2 and D3. Clin Chem. 2005;51(9):1683-1690. doi:10.1373/clinchem.2005.052936

4. Cantio E, Bilenberg N, Nørgaard SM, et al. Vitamin D status in pregnancy and childhood associates with intelligence quotient at age 7 years: An Odense child cohort study. Aust N Z J Psychiatry. 2023;57(7):1062-1072. doi:10.1177/00048674221116027

5.Darling AL, Rayman MP, Steer CD, Golding J, Lanham-New SA, Bath SC. Association between maternal vitamin D status in pregnancy and neurodevelopmental outcomes in childhood: results from the Avon Longitudinal Study of Parents and Children (ALSPAC). Br J Nutr. 2017;117(12):1682-1692. doi:10.1017/S0007114517001398

6.Keim SA, Bodnar LM, Klebanoff MA. Maternal and cord blood 25(OH)-vitamin D concentrations in relation to child development and behaviour. Paediatr Perinat Epidemiol. 2014;28(5):434-444. doi:10.1111/ppe.12135

7.Kuzmickienė J, Kaubrys G. Selective Ability of Some CANTAB Battery Test Measures to Detect Cognitive Response to a Single Dose of Donepezil in Alzheimer Disease. Med Sci Monit Int Med J Exp Clin Res. 2015;21:2572-2582. doi:10.12659/MSM.895381

8.Weschler D. WISC-IV Wechsler Intelligence Scale for Children - Fourth Edition. Vejledning Del 1 Dansk Version.; 2010.

9.Sahakian BJ, Owen AM. Computerized assessment in neuropsychiatry using CANTAB: discussion paper. J R Soc Med. 1992;85(7):399-402.

10.Cambridge Cognition. Product Overview: CANTAB Connect Research.; 2018.

11.Reynolds CR, Voss JK. Test Af Hukommelse Og Indlæring 2. Udgave - Vejledning. 1st ed.; 2017.

12.Weschler D. WISC-IV Technical and Interpretive Manual.; 2003.

13.Mena-Bravo A, Calderón-Santiago M, Lope V, et al. Vitamin D3 levels in women and factors contributing to explain metabolic variations. J Steroid Biochem Mol Biol. 2021;211:105884. doi:10.1016/j.jsbmb.2021.105884

14.Rajan S, Weishaar T, Keller B. Weight and skin colour as predictors of vitamin D status: results of an epidemiological investigation using nationally representative data. Public Health Nutr. 2017;20(10):1857-1864. doi:10.1017/S1368980016000173

15.Horner D, Jepsen JRM, Chawes B, et al. A western dietary pattern during pregnancy is associated with neurodevelopmental disorders in childhood and adolescence. Nat Metab. 2025;7(3):586-601. doi:10.1038/s42255-025-01230-z

16.Mohammadzadeh P, Jepsen JRM, Lemvigh CK, et al. Maternal interleukin 6 in pregnancy is associated with everyday, but not test-based executive functioning in 10-year-old children. Psychol Med. 2025;55:e112. doi:10.1017/S0033291725000674

17.Eves R, Wolke D, Spiegler J, Lemola S. Association of Birth Weight Centiles and Gestational Age With Cognitive Performance at Age 5 Years. JAMA Netw Open. 2023;6(8):e2331815.


© 2026 Frederiksen OF et al. *JAMA Network Open.*

doi:10.1001/jamanetworkopen.2023.31815

18.Hansen L, Tjønneland A, Køster B, et al. Vitamin D Status and Seasonal Variation among Danish Children and Adults: A Descriptive Study. Nutrients. 2018;10(11):1801. doi:10.3390/nu10111801

19.Horta BL, Loret De Mola C, Victora CG. Breastfeeding and intelligence: a systematic review and meta-analysis. Acta Paediatr. 2015;104(S467):14-19. doi:10.1111/apa.13139

20.Du J, Rolls ET, Gong W, et al. Association between parental age, brain structure, and behavioral and cognitive problems in children. Mol Psychiatry. 2022;27(2):967-975. doi:10.1038/s41380-021-01325-5

© 2026 Frederiksen OF et al. *JAMA Network Open.*

**eTable 1.** Overview of Covariates for the Adjusted Main and Secondary Analyses

| **Main RCT analyses** | **Observational week 24 analyses** |
|---|---|
| *Factor variables* | *Factor variables* |
| Child sex | Child sex |
| Season of birth | Season of 25(OH)D measurement |
| N-3-LCPUFA intervention status | Gestational diabetes mellitus |
| | Preeclampsia |
| | Smoking during pregnancy |
| | Alcohol during pregnancy |
| | Maternal education |
| | Household income |
| | |
| *Continuous variables* | *Continuous variables* |
| Maternal pre-intervention 25(OH)D level | Birthweight |
| Age at COPSYCH visit | Gestational age |
| | Maternal pre-pregnancy BMI |
| | Maternal pregnancy inflammation (IL6, CRP) |
| | Maternal pregnancy diet |
| | Maternal age |
| | Paternal age |
| | Age at COPSYCH visit |

© 2026 Frederiksen OF et al. *JAMA Network Open.*

**eTable 2.** Baseline Characteristics of Participants Included in the RCT and the COPSYCH Project

| | Overall<br>N = 498 | Vitamin D<br>N = 247 | Placebo<br>N = 251 |
|---|---|---|---|
| Maternal BMI, mean (SD) | 24.7 (4.6) | 24.5 (4.6) | 24.9 (4.5) |
| Maternal age, y, mean (SD) | 32.4 (4.3) | 32.7 (4.4) | 32.0 (4.2) |
| Pre-interventional 25(OH)D level, nmol, median [IQR] | 75.6 [58.2, 92.2] | 76.1 [58.5, 92.6] | 75.5 [58.1, 91.1] |
| Pre-interventional 25(OH)D level, >75 nmol/L (30 ng/mL), N (%) | 253 (51.2) | 128 (52.2) | 125 (50.2) |
| Pre-interventional 25(OH)D level, >100 nmol/L, (40ng/mL) N (%) | 83 (16.7) | 41 (16.6) | 42 (16.7) |
| Post-interventional 25(OH)D level, nmol, median [IQR] | 86.7 [62.5, 114.5] | 104.5 [86.2, 127.3] | 68.7 [46.1, 90.1] |
| Post-interventional 25(OH)D level, >75 nmol/L (30 ng/mL), N (%) | 313 (63.7) | 205 (84.0) | 108 (43.7) |
| Post-interventional 25(OH)D level, >100 nmol/L (40 ng/mL), N (%) | 184 (36.9) | 142 (57.5) | 42 (16.7) |
| Unhealthy diet PC, mean (SD)[1] | 0.04 (1.01) | -0.04 (0.94) | 0.1 (1.1) |
| Maternal IL6, median [IQR] | 0.3 [0.2, 0.4] | 0.3 [0.2, 0.4] | 0.3 [0.2, 0.4] |
| Maternal CRP, median [IQR] | 5.1 [2.5, 10.2] | 5.2 [2.6, 10.0] | 5.1 [2.5, 10.3] |
| Maternal education | | | |
| Elementary | 18 (3.6) | 7 (2.8) | 11 (4.4) |
| High School | 23 (4.6) | 10 (4.0) | 13 (5.2) |
| Tradesman | 100 (20.1) | 42 (17.0) | 58 (23.1) |
| Bachelor's degree | 215 (43.2) | 110 (44.5) | 105 (41.8) |
| Master's degree | 142 (28.5) | 78 (31.6) | 64 (25.5) |
| Household income, N (%)[2] | | | |
| <100.000 DKK | 44 (8.8) | 21 (8.5) | 23 (9.2) |
| 100.000-150.000 DKK | 105 (21.1) | 48 (19.4) | 57 (22.7) |
| 150.000-200.000 DKK | 154 (30.9) | 76 (30.8) | 78 (31.1) |
| 200.000-250.000 DKK | 121 (24.3) | 61 (24.7) | 60 (23.9) |
| >250.000 DKK | 74 (14.9) | 41 (16.6) | 33 (13.1) |
| Alcohol use during pregnancy, yes, N (%)[3] | 81 (16.3) | 42 (17.1) | 39 (15.5) |
| Smoking during pregnancy, yes, N (%)[3] | 37 (7.4) | 16 (6.5) | 21 (8.4) |
| Parity, N (%) | | | |
| 1 | 221 (44.4) | 97 (39.3) | 124 (49.4) |
| 2 | 198 (39.8) | 107 (43.3) | 91 (36.3) |
| >2 | 79 (15.9) | 43 (17.4) | 36 (14.3) |
| n3-LCPUFA intervention, yes, N(%) | 249 (50.0) | 127 (51.4) | 122 (48.6) |
| Preeclampsia, yes, N (%) | 25 (5.0) | 13 (5.3) | 12 (4.8) |
| | **Overall** | **Vitamin D** | **Placebo** |

© 2026 Frederiksen OF et al. *JAMA Network Open.*

| | | | |
|---|---|---|---|
| Antibiotics during pregnancy, yes, N (%) | 171 (34.4) | 82 (33.3) | 89 (35.5) |
| Gestational diabetes mellitus, yes, N (%) | 7 (1.4) | 2 (0.8) | 5 (2.0) |
| Paternal age, y, mean (SD) | 34.6 (5.2) | 35.0 (5.2) | 34.3 (5.2) |
| Paternal education, N (%) | | | |
| Elementary | 22 (4.5) | 7 (2.9) | 15 (6.1) |
| High School | 25 (5.2) | 13 (5.4) | 12 (4.9) |
| Tradesman | 150 (30.9) | 69 (28.9) | 81 (32.9) |
| Bachelor's degree | 146 (30.1) | 76 (31.8) | 70 (28.5) |
| Master's degree | 142 (29.3) | 74 (31.0) | 68 (27.6) |
| Birthweight, kg, mean (SD) | 3.55 (0.53) | 3.56 (0.55) | 3.53 (0.50) |
| Gestational age at birth, days, mean (SD) | 279.4 (10.9) | 279.5 (11.5) | 279.4 (10.3) |
| Sex, female, N (%) | 240 (48) | 113 (46) | 127 (51) |
| Sex, male, N (%) | 258 (52) | 134 (54.3) | 124 (49.4) |
| Season of birth, N (%) | | | |
| Winter | 180 (36.1) | 93 (37.7) | 87 (34.7) |
| Spring | 98 (19.7) | 47 (19.0) | 51 (20.3) |
| Summer | 100 (20.1) | 50 (20.2) | 50 (19.9) |
| Fall | 120 (24.1) | 57 (23.1) | 63 (25.1) |
| Race[4], non-white, N (%) | 22 (4) | 11 (4.5) | 11 (4.4) |
| Race[4], white, N (%) | 476 (96) | 236 (96) | 240 (96) |
| Solely breastfed, days, median [IQR] | 122 [60, 151] | 122 [56.5, 149.5] | 123.00 [64, 151.5] |
| ADHD, yes, N (%) | 58 (11.7) | 27 (11.0) | 31 (12.4) |
| ASD, yes, N (%) | 12 (2.4) | 5 (2.0) | 7 (2.8) |

Abbreviations: SD, Standard deviation; IQR, interquartile range; N, number; ADHD, attention-deficit/hyperactivity disorder; ASD, autism spectrum disorder
[1]Unhealthy diet represents a Western dietary pattern identified via PCA in [Horner D, Nature Metabolism, 2025
[2]Income is self-reported combined household income 3 months before birth]
[3]Alcohol and smoking during pregnancy reflects use at any time during pregnancy
[4]Information on race was obtained through parental interviews and was defined as either white or non-white

© 2026 Frederiksen OF et al. *JAMA Network Open.*

**eTable 3.** Mean Values of all Tests Included in the Cognitive Test Battery From the COPSYCH Visit at Age 10

| | Vitamin D | | Placebo | | |
|---|---|---|---|---|---|
| **Test** | **N** | **Mean (SD)** | **N** | **Mean (SD)** | **Outcome metric** |
| Estimated Intelligence | 245 | 107.6 (15) | 250 | 107.8 (13.2) | Index score |
| Matrices | 246 | 23.5 (3.9) | 251 | 23.4 (3.8) | Total number correct |
| Vocabulary | 246 | 29.5 (6.3) | 250 | 29.6 (5.6) | Total number correct |
| Symbol Search | 247 | 23.6 (4.7) | 250 | 23.8 (4.5) | Sum of total number minus errors |
| Coding | 247 | 37.7 (7.8) | 251 | 37.8 (8) | Total number correct |
| Reaction Time, simple | 246 | 364.2 (50.3) | 249 | 369.5 (45.9) | milliseconds |
| Reaction Time, five-choice | 247 | 412.8 (57.6) | 249 | 419.7 (57.2) | milliseconds |
| Rapid Visual Information Processing | 247 | 0.8 (0.1) | 248 | 0.8 (0.1) | A' |
| Movement Time, simple | 246 | 219.1 (62.7) | 249 | 223.9 (73) | milliseconds |
| Movement Time, five-choice | 247 | 240.5 (52.9) | 249 | 246.3 (54.9) | milliseconds |
| Word Selective Reminding | 245 | 56.9 (7) | 250 | 55.7 (7.4) | Total number recalled |
| Object Recall | 246 | 51.7 (8.9) | 250 | 50.6 (8.7) | Total number recalled |
| Paired Associates Learning | 247 | 11.1 (9.8) | 250 | 13.9 (13.1) | Total errors |
| Letter-Number Sequencing | 247 | 16.5 (2.8) | 251 | 16.8 (2.8) | Total number correct |
| Digit Span | 247 | 14 (2.5) | 251 | 14.3 (2.4) | Total number correct |
| Intra Extra Dimensional Set Shift | 244 | 15.4 (10.6) | 251 | 17.5 (10) | Extra-dimensional errors |
| Spatial Working Memory | 247 | 11.4 (7.9) | 251 | 10.5 (7.5) | Total errors |
| Stockings of Cambridge | 247 | 7.3 (2) | 250 | 7.3 (1.8) | Problems solved in minimum moves |

© 2026 Frederiksen OF et al. *JAMA Network Open.*

**eTable 4.** Interaction Analyses of Vitamin D3 Supplementation With Sex, Maternal Week 24 25(OH)D Level, Offspring 6 Months and 6 Year 25(OH)D Levels and N-3-LCPUFA Intervention

| Function | Week 24* p-value | 6 month** p-value | 6 year*** p-value | Sex p-value | N-3-LCPUFA p-value |
|---|---|---|---|---|---|
| Estimated Intelligence | 0.24 | 0.72 | 0.34 | 0.26 | 0.35 |
| Speed of processing | 0.79 | 0.06 | 0.73 | 0.20 | 0.27 |
| Reaction time | 0.42 | 0.21 | 0.43 | 0.44 | 0.94 |
| Sustained attention | 0.55 | 0.39 | 0.76 | 0.92 | 0.43 |
| Motor speed | **0.04** | 0.48 | 0.84 | 0.98 | 0.65 |
| Verbal memory | 0.12 | 0.51 | 0.74 | 0.76 | 0.82 |
| Verbal working memory | 0.62 | 0.66 | 0.58 | 1.00 | 0.06 |
| Visual memory | 0.92 | 0.75 | 0.73 | 0.72 | 0.76 |
| Flexibility/Set shift | 0.30 | 0.06 | 0.95 | 0.57 | 0.35 |
| Spatial Working Memory | **0.02** | 0.26 | 0.10 | 0.34 | 0.27 |
| Planning | 0.18 | **0.04** | 0.44 | 0.37 | 0.92 |

*maternal pre-interventional 25(OH)D level
**25(OH)D level in offspring at 6 months of age
***25(OH)D level in offspring at 6 years of age

© 2026 Frederiksen OF et al. *JAMA Network Open.*

**eTable 5.** Effect of High-Dose vs Standard-Dose Vitamin D3 Supplementation During Pregnancy on the Cognitive Functions at Age 10, Split by Children With and Without an ADHD Diagnosis, Including P-Interaction

| | Without ADHD | | | | With ADHD | | | | |
|---|---|---|---|---|---|---|---|---|---|
| **Function** | **N** | **Estimate [CI]** | **p-value** | **q-value*** | **N** | **Estimate [CI]** | **p-value** | **q-value*** | **p-interaction**** |
| Estimated Intelligence | 433 | 0.01 [-0.17;0.20] | 0.87 | 0.87 | 56 | -0.22 [-0.77;0.33] | 0.43 | 0.43 | 0.33 |
| Speed of Processing | 435 | -0.03 [-0.19;0.12] | 0.68 | 0.68 | 57 | -0.06 [-0.59;0.47] | 0.82 | 0.82 | 0.91 |
| Reaction Time | 435 | 0.09 [-0.08;0.27] | 0.28 | 0.28 | 55 | 0.31 [-0.22;0.83] | 0.24 | 0.24 | 0.68 |
| Sustained Attention | 435 | -0.01 [-0.19;0.17] | 0.93 | 0.93 | 54 | -0.11 [-0.82;0.60] | 0.76 | 0.76 | 0.48 |
| Motor Speed | 435 | 0.07 [-0.11;0.24] | 0.45 | 0.45 | 55 | 0.31 [-0.37;0.98] | 0.37 | 0.37 | 0.58 |
| Verbal Memory | 435 | 0.18 [0.03;0.33] | **0.02** | **0.02** | 56 | 0.08 [-0.40;0.57] | 0.73 | 0.82 | 0.81 |
| Verbal Working Memory | 435 | -0.13 [-0.28;0.02] | 0.09 | 0.09 | 57 | 0.08 [-0.45;0.60] | 0.77 | 0.77 | 0.47 |
| Visual Memory | 435 | 0.28 [0.09;0.48] | **0.01** | **0.01** | 56 | -0.07 [-0.70;0.56] | 0.82 | 0.82 | 0.24 |
| Flexibility/Set shift | 434 | 0.16 [-0.02;0.35] | 0.09 | 0.26 | 55 | 0.50 [-0.09;1.10] | 0.10 | 0.14 | 0.30 |
| Spatial Working Memory | 435 | -0.09 [-0.28;0.10] | 0.33 | 0.46 | 57 | -0.48 [-1.04;0.07] | 0.09 | 0.14 | 0.48 |
| Planning | 435 | 0.07 [-0.11;0.25] | 0.46 | 0.46 | 56 | -0.37 [-0.93;0.19] | 0.19 | 0.19 | 0.07 |

ADHD = attention-deficit/hyperactivity disorder
Results adjusted for sex. age at testing. PUFA intervention. season of birth and pre-interventional 25(OH)D level
*Benjamini-Hochberg false discovery rate (5%) applied across domains, domains comprising a single function were not subject to multiplicity correction; q < 0.05 considered significant
**Interaction analyses from linear regression models of vitamin D3 intervention x ADHD diagnosis

© 2026 Frederiksen OF et al. *JAMA Network Open.*

**eTable 6.** Effect of High-Dose vs Standard-Dose Vitamin D3 Supplementation During Pregnancy on the Cognitive Functions at Age 10, Split by Children With and Without an ASD Diagnosis, Including P-Interaction

| | Without ASD | | | | With ASD | | | | |
|---|---|---|---|---|---|---|---|---|---|
| **Function** | **n** | **Estimate (CI)** | **p-value** | **q-value*** | **n** | **Estimate (CI)** | **p-value** | **q-value*** | **p-interaction**** |
| Estimated Intelligence | 477 | 0 [-0.18;0.18] | 0.99 | 0.99 | 12 | 0 [-5.13;5.13] | 1 | 1 | 0.51 |
| Speed of Processing | 480 | -0.04 [-0.20;0.11] | 0.58 | 0.58 | 12 | 0.49 [-2.57;3.54] | 0.65 | 0.65 | 0.19 |
| Reaction Time | 479 | 0.08 [-0.09;0.24] | 0.37 | 0.37 | 11 | 4.6 [-1.17;10.38] | 0.09 | 0.09 | **0.01** |
| Sustained Attention | 479 | -0.03 [-0.22;0.15] | 0.73 | 0.73 | 10 | 2.96 [-2.00;7.92] | 0.12 | 0.12 | 0.28 |
| Motor Speed | 479 | 0.07 [-0.10;0.24] | 0.40 | 0.40 | 11 | 3.7 [-1.37;8.77] | 0.10 | 0.10 | 0.30 |
| Verbal Memory | 479 | 0.18 [0.03;0.32] | **0.02** | **0.02** | 12 | 0.11 [-3.46;3.68] | 0.93 | 0.93 | 0.52 |
| Verbal Working Memory | 480 | -0.11 [-0.26;0.04] | 0.15 | 0.15 | 12 | 0.87 [-2.62;4.36] | 0.49 | 0.49 | 0.53 |
| Visual Memory | 479 | 0.22 [0.03;0.41] | **0.02** | **0.02** | 12 | -0.98 [-5.50;3.55] | 0.54 | 0.93 | 0.31 |
| Flexibility/Set shift | 477 | 0.18 [0.00;0.36] | **0.045** | 0.14 | 12 | 1.19 [-2.04;4.42] | 0.33 | 0.40 | 0.34 |
| Spatial Working Memory | 480 | -0.13 [-0.31;0.05] | 0.17 | 0.25 | 12 | -1.1 [-4.69;2.49] | 0.40 | 0.40 | 0.44 |
| Planning | 479 | 0.04 [-0.13;0.22] | 0.65 | 0.65 | 12 | -2.45 [-5.84;0.95] | 0.11 | 0.32 | **0.03** |

Adjusted for sex, age at testing, PUFA intervention, season of birth and pre-interventional 25(OH)D level
ASD = autism spectrum disorder
*Benjamini-Hochberg false discovery rate (5%) applied across domains, domains comprising a single function were not subject to multiplicity correction; q < 0.05 considered significant
**Interaction analyses from linear regression models of vitamin D3 intervention x ASD diagnosis

© 2026 Frederiksen OF et al. *JAMA Network Open.*

**eTable 7.** Effect of High-Dose vs Standard-Dose Vitamin D3 Supplementation on All the Tests Included in the Neurocognitive Test Battery

| | Crude | | | Adjusted* | | | |
|---|---|---|---|---|---|---|---|
| **Test** | **N** | **Estimate [CI]** | **p-value** | **N** | **Estimate [CI]** | **p-value** | **q-value**** |
| Matrices | 497 | 0.03 [-0.14;0.21] | 0.71 | 493 | 0.04 [-0.13;0.21] | 0.65 | 0.75 |
| Vocabulary | 496 | -0.02 [-0.2;0.15] | 0.80 | 492 | 0.03 [-0.2;0.14] | 0.75 | 0.75 |
| Coding | 498 | -0.01 [-0.19;0.16] | 0.87 | 494 | 0.01 [-0.16;0.18] | 0.94 | 0.94 |
| Symbol search | 497 | -0.05 [-0.23;0.12] | 0.55 | 493 | 0.04 [-0.22;0.13] | 0.64 | 0.94 |
| Reaction time, simple | 495 | 0.11 [-0.07;0.29] | 0.22 | 491 | 0.11 [-0.07;0.3] | 0.22 | 0.22 |
| Reaction time, five-choice | 496 | 0.12 [-0.06;0.3] | 0.18 | 492 | 0.11 [-0.07;0.29] | 0.21 | 0.22 |
| Rapid visual information processing | 495 | -0.03 [-0.21;0.15] | 0.77 | 491 | -0.03 [-0.21;0.16] | 0.79 | 0.79 |
| Movement time, simple | 495 | 0.08 [-0.11;0.26] | 0.42 | 491 | 0.07 [-0.12;0.25] | 0.49 | 0.49 |
| Movement time, five-choice | 496 | 0.11 [-0.07;0.29] | 0.23 | 492 | 0.09 [-0.08;0.26] | 0.31 | 0.49 |
| Word selective reminding | 495 | 0.17 [0;0.34] | 0.06 | 491 | 0.2 [0.03;0.37] | **0.02** | **0.03** |
| Object recall | 496 | 0.13 [-0.05;0.3] | 0.15 | 492 | 0.15 [-0.02;0.31] | 0.09 | 0.09 |
| Paired associates, total errors | 497 | 0.25 [0.07;0.44] | **0.01** | 493 | 0.24 [0.06;0.42] | **0.01** | **0.03** |
| Digit span | 498 | -0.12 [-0.29;0.05] | 0.17 | 494 | -0.12 [-0.29;0.06] | 0.19 | 0.31 |
| Letter number sequencing | 498 | -0.09 [-0.26;0.08] | 0.30 | 494 | -0.09 [-0.26;0.08] | 0.31 | 0.31 |
| Intra extra dimensional set shift | 495 | 0.20 [0.02;0.38] | **0.03** | 491 | 0.19 [0.01;0.37] | **0.03** | 0.09 |
| Spatial working memory | 498 | -0.11 [-0.29;0.06] | 0.20 | 494 | -0.12 [-0.3;0.06] | 0.18 | 0.27 |
| Stockings of Cambridge | 497 | 0 [-0.18;0.17] | 0.96 | 493 | 0 [-0.17;0.17] | 0.98 | 0.98 |

All estimates are based on standardized z-scores; higher values indicate better performance.
*Adjusted for sex. age at testing. PUFA intervention. season of birth and pre-interventional 25(OH)D level
**Benjamini-Hochberg false discovery rate (5%) applied across domains, domains comprising a single function were not subject to multiplicity correction; q < 0.05 considered significant.

© 2026 Frederiksen OF et al. *JAMA Network Open.*

**eTable 8.** Achieved-Level Analysis of Maternal Postpartum 25(OH)D (≥100 vs <100 nmol/L) and Offspring Cognitive Outcomes

| Domain | Outcome | N | Estimate (CI) | p-value | N | Estimate (CI), adjusted* | p-value | q-value** |
|---|---|---|---|---|---|---|---|---|
| Estimated Inteligence | Estimated Intelligence | 491 | -0.00 (-0.24, 0.24) | 1.00 | 491 | 0.04 (-0.28, 0.37) | 0.79 | 0.79 |
| Processing Speed | Speed of Processing | 494 | -0.03 (-0.24, 0.18) | 0.79 | 494 | -0.04 (-0.32, 0.24) | 0.79 | 0.79 |
| Reaction Time | Reaction Time | 492 | 0.11 (-0.11, 0.33) | 0.32 | 492 | 0.22 (-0.08, 0.52) | 0.16 | 0.16 |
| Attention | Sustained Attention | 491 | -0.15 (-0.39, 0.09) | 0.21 | 491 | -0.28 (-0.61, 0.05) | 0.10 | 0.10 |
| Motor Function | Motor Speed | 492 | 0.05 (-0.18, 0.28) | 0.66 | 492 | -0.04 (-0.35, 0.27) | 0.80 | 0.80 |
| Memory | Verbal Memory | 493 | 0.11 (-0.09, 0.31) | 0.30 | 493 | 0.10 (-0.17, 0.37) | 0.47 | 0.64 |
| Working Memory | Verbal Working Memory | 494 | 0.00 (-0.19, 0.20) | 0.98 | 494 | -0.13 (-0.40, 0.14) | 0.34 | 0.34 |
| Memory | Visual Memory | 493 | 0.02 (-0.22, 0.27) | 0.86 | 493 | -0.08 (-0.42, 0.26) | 0.64 | 0.64 |
| Executive Function | Flexibility/Set Shift | 491 | **0.30 (0.06, 0.54)** | **0.01** | 491 | 0.24 (-0.09, 0.57) | 0.15 | 0.22 |
| Executive Function | Spatial Working Memory | 494 | -0.03 (-0.27, 0.20) | 0.78 | 494 | -0.09 (-0.41, 0.24) | 0.61 | 0.61 |
| Executive Function | Planning | 493 | -0.14 (-0.38, 0.09) | 0.22 | 493 | -0.24 (-0.55, 0.08) | 0.15 | 0.22 |

Exposure defined by achieved maternal postpartum 25(OH)D concentration, comparing participants who reached ≥100 nmol/L with those who did not
*Adjusted for sex, age at testing, PUFA intervention, season of birth and pre-interventional 25(OH)D level
**Benjamini-Hochberg false discovery rate (5%) applied across domains, domains comprising a single function were not subject to multiplicity correction; q < 0.05 considered significant

© 2026 Frederiksen OF et al. *JAMA Network Open.*

**eFigure 1.** COPSAC2010 and COPSYCH Measures

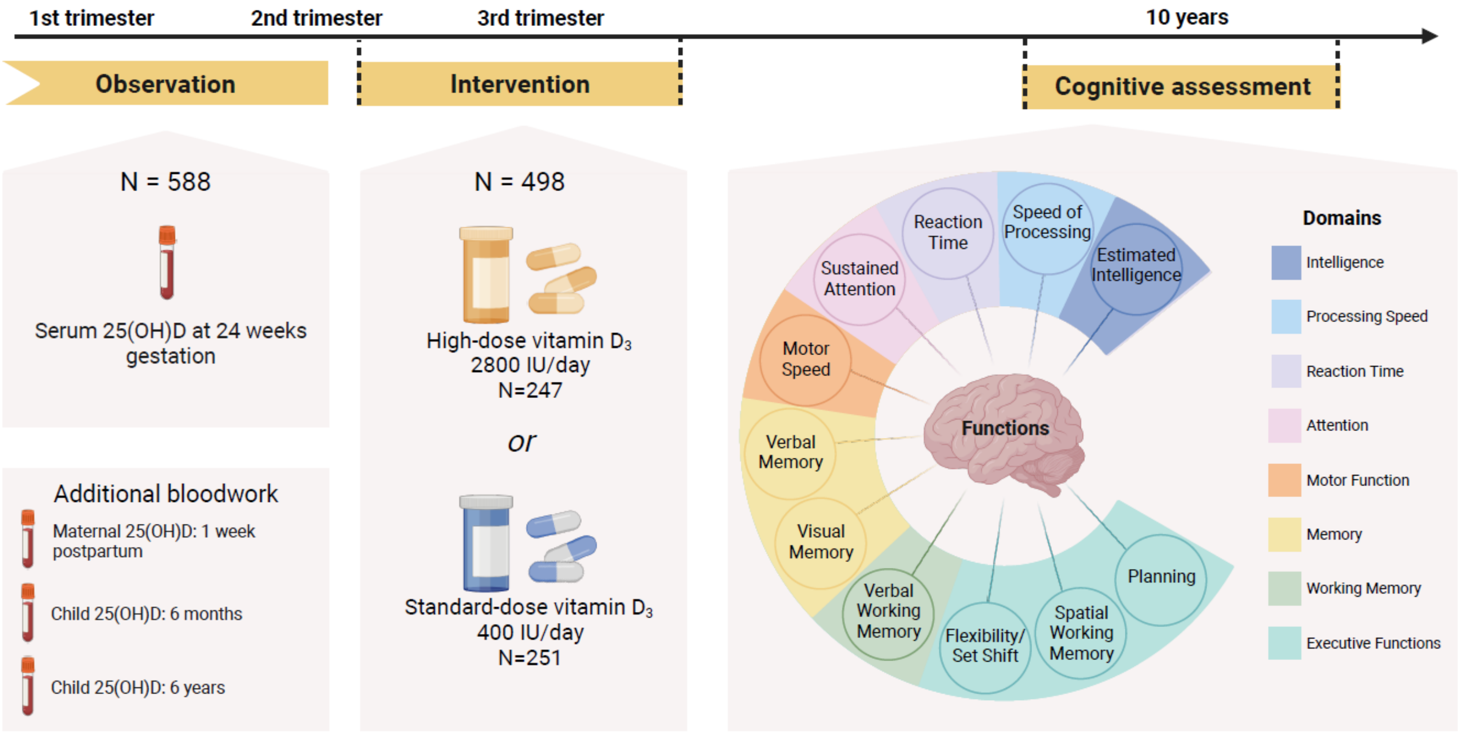


Figure 1 was created in BioRender. Bønnelykke, K. (2026) https://BioRender.com/mz5eb6h.

© 2026 Frederiksen OF et al. *JAMA Network Open.*

**eFigure 2.** Directed Acyclic Graph: Pre-Interventional 25(OH)D Levels and Childhood Cognition

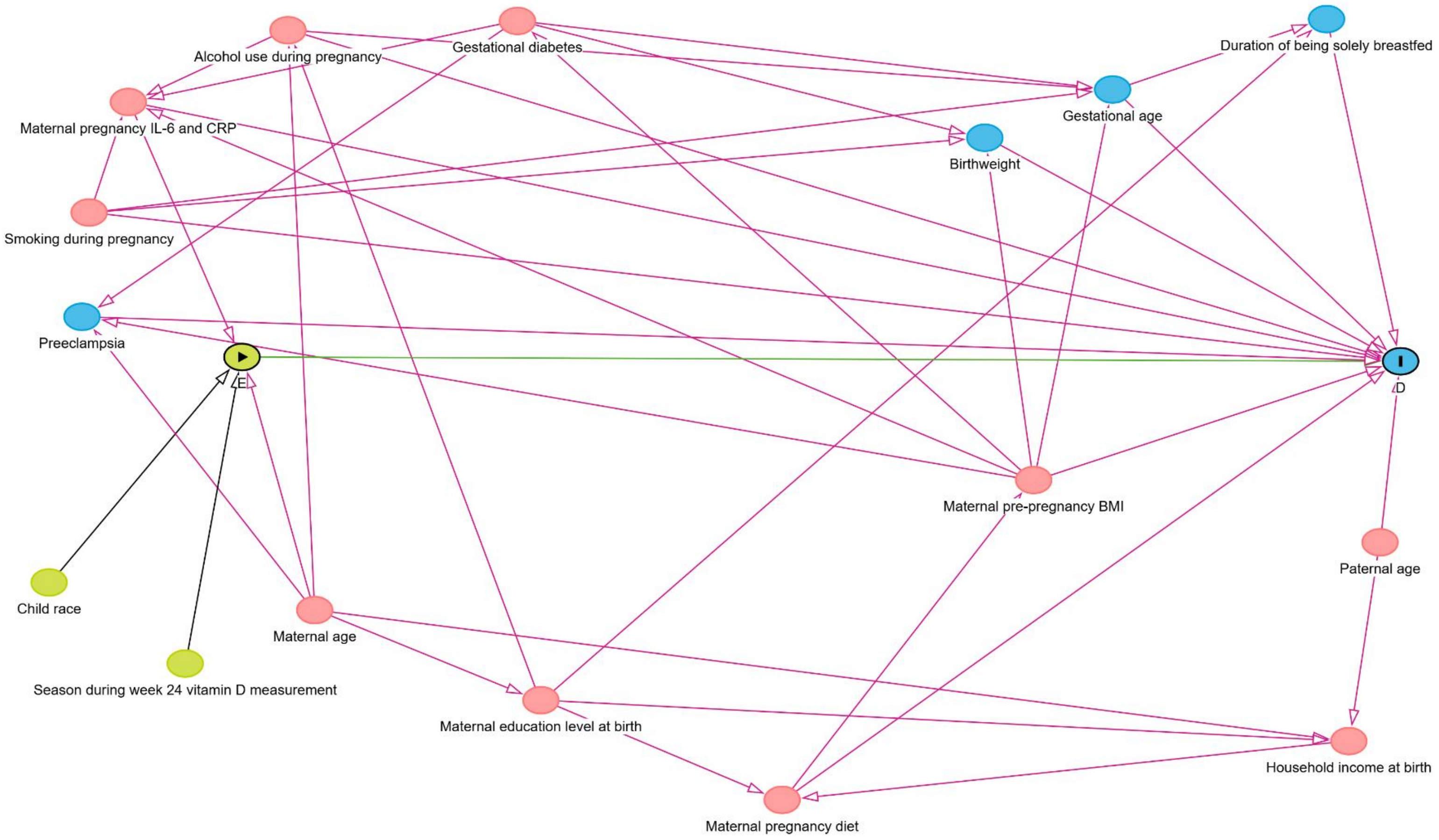


**Created using:** https://www.dagitty.net/dags.html#

© 2026 Frederiksen OF et al. *JAMA Network Open.*

**eFigure 3.** Heatmap: Spearman Correlation Between All Cognitive Tests From the Original COPSYCH Protocol

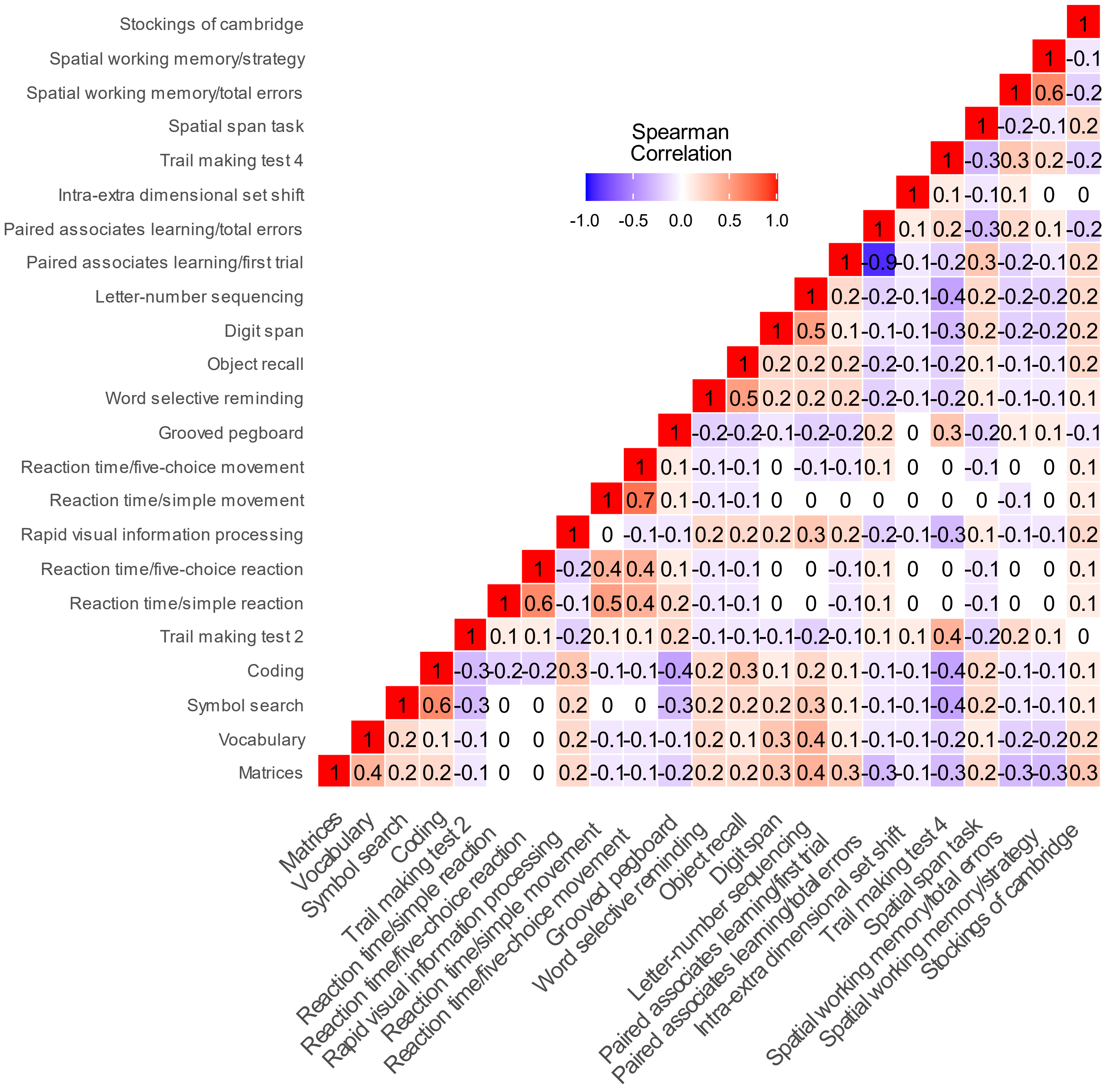


© 2026 Frederiksen OF et al. *JAMA Network Open.*

**eFigure 4.** Violin Plot: Change in Maternal 25(OH)D levels From Pre- to Post-Intervention

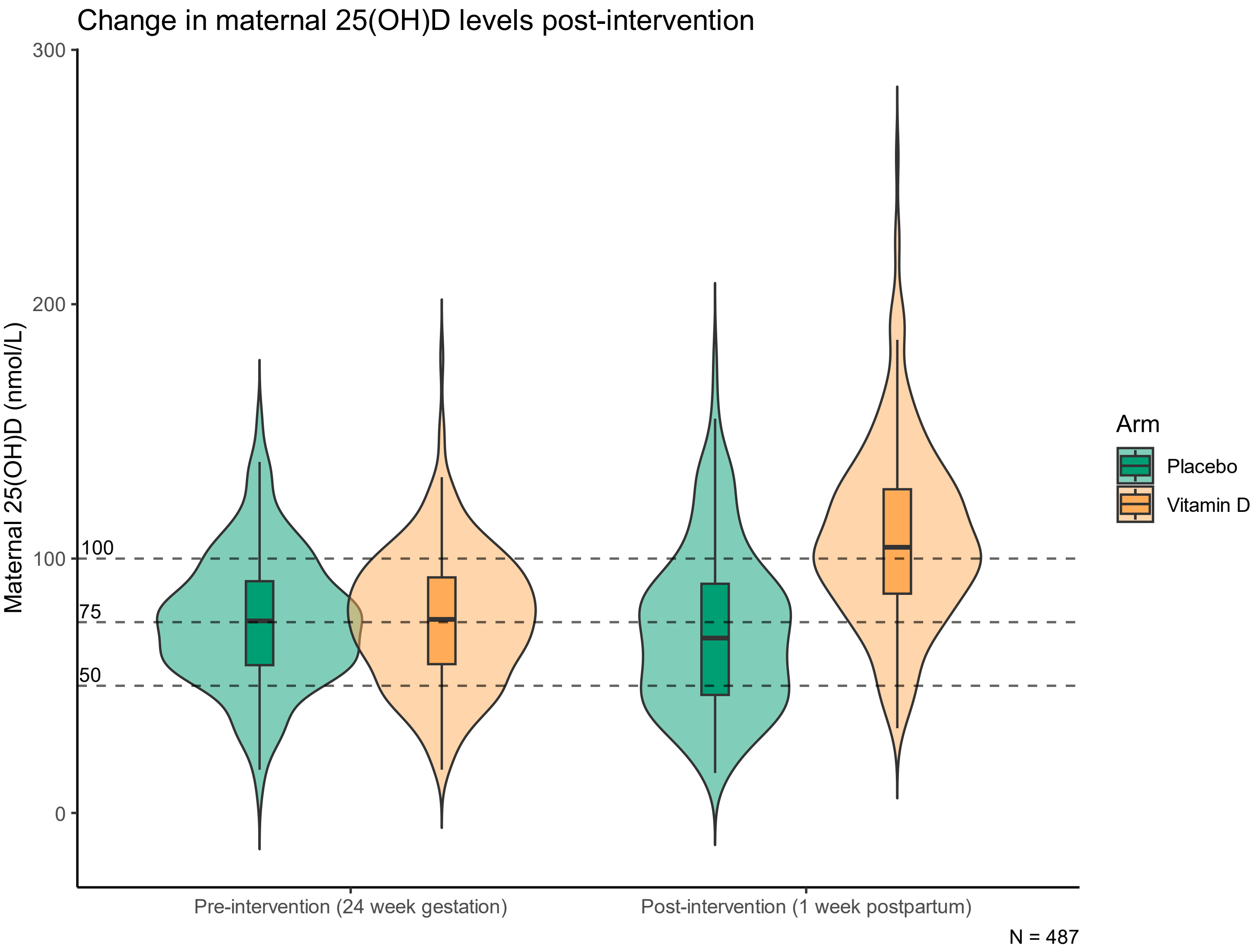


Observed maternal 25(OH)D concentrations ranged from 7.8-179 nmol/L at gestation week 24 and from 15.7-258 at week 1 postpartum.

The violin plot shows change in maternal 25(OH)D (nmol/L) from gestational week 24 to 1 week postpartum by vitamin D3 supplementation group (standard-dose 400IU/day vs high-dose (2800IU/day). The central line indicates the median and the box indicates the interquartile range. Violin width reflects data density.

© 2026 Frederiksen OF et al. *JAMA Network Open.*

**eFigure 5.** Forest Plot of Effect of Vitamin D3 Supplementation on Cognitive Functions Including q-Values

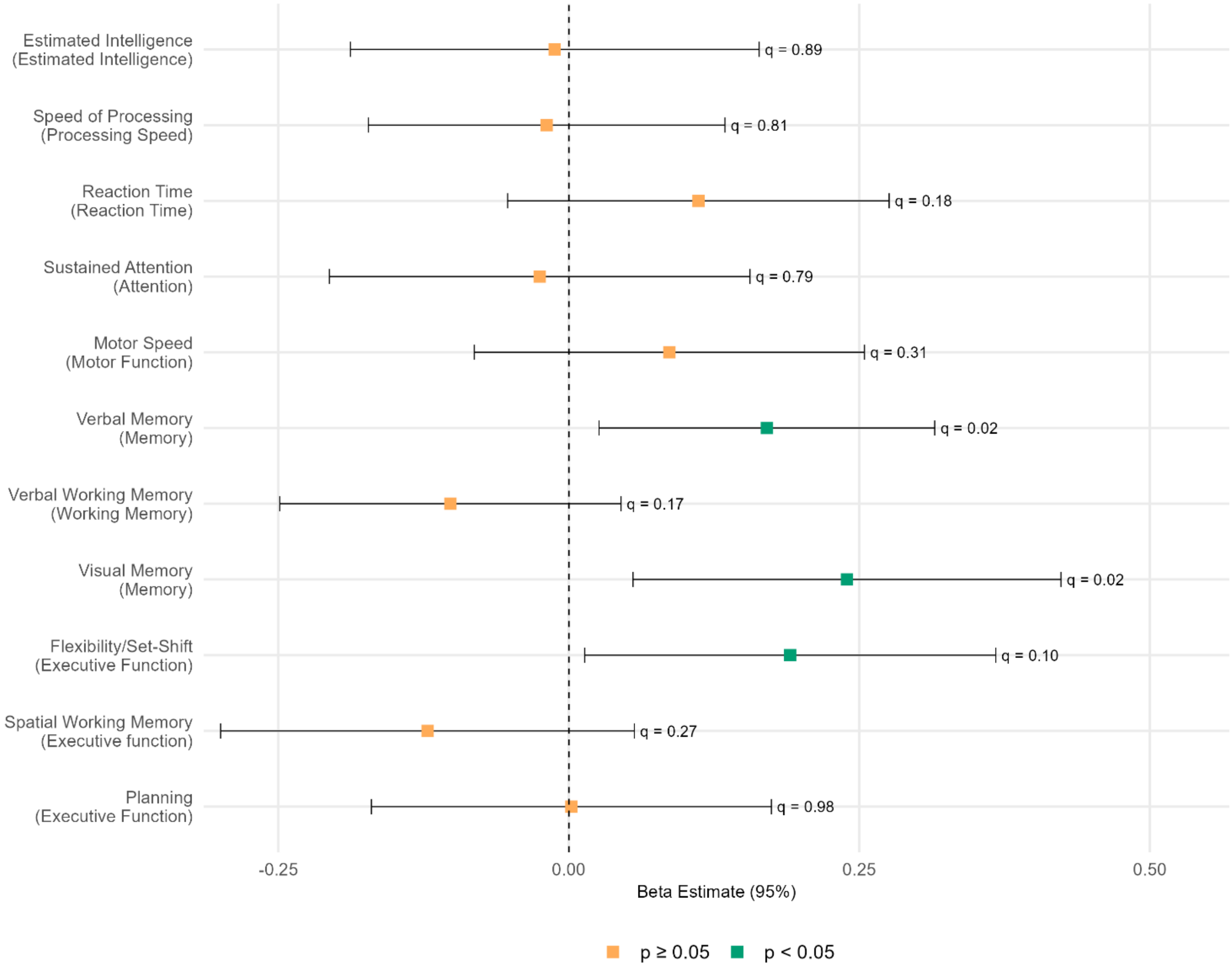


Corresponding domains are listed in parenteses under each function
Analyses are adjusted for sex, age at COPSYCH visit, n-3 LCPUFA intervention, season of birth, and 25(OH)D at week 24.
Statistical significance was reported according to nominal p-values
Benjamini-Hochberg false discovery rate (5%) applied across domains, domains comprising a single function were not subject to multiplicity correction, $q < 0.05$ considered significant

© 2026 Frederiksen OF et al. *JAMA Network Open.*

**eFigure 6.** Restricted Cubic Spline Showing the Association Between Maternal Postpartum 25(OH)D Concentration and Cognitive Outcomes

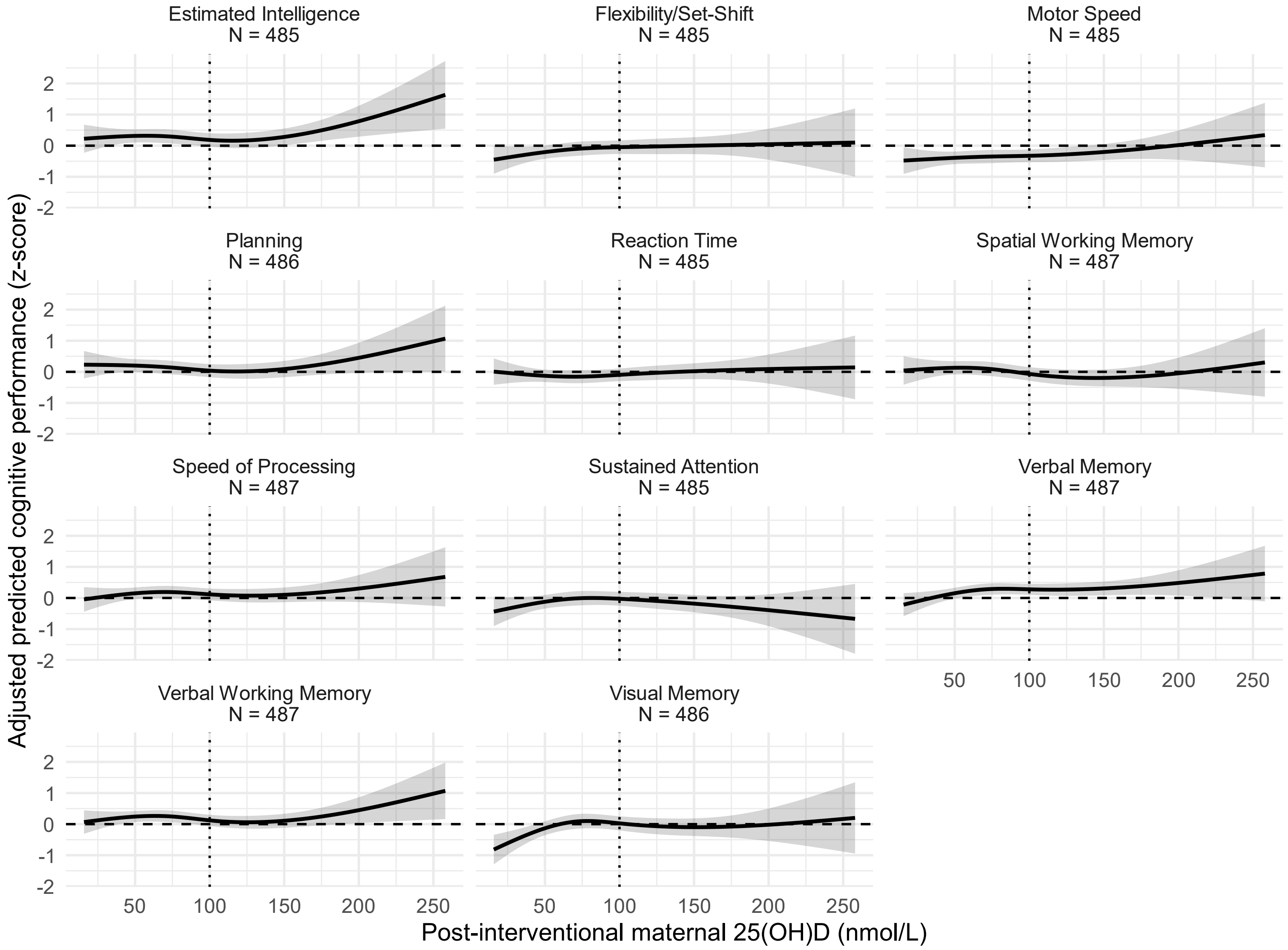


Dose-response association between post-interventional maternal 25(OH)D at 1 week postpartum and offspring cognition outcomes (z-scores) at age 10. Curves show covariate adjusted predicted mean z-scores from linear regression models including a natural cubic spline for post-interventional 25(OH)D (3 degrees of freedom). Shaded areas indicate 95% confidence intervals. The dashed horizontal line marks z=0 and the dotted vertical line marks 100 nmol/L.
Adjusted for child sex, age at COPSYCH visit, n-3-LCPUFA intervention group, birthdate season and maternal 25(OH)D level at 24 weeks gestation.
Interpretation at high levels of 25(OH)D should be cautious due to fewer observations at the upper end of the exposure distribution (N with 25(OH)D > 150 nmol/L = 34)
Created using R version 4.3.1

© 2026 Frederiksen OF et al. *JAMA Network Open.*
