# Supplementary material for: High-Dose Vitamin D3 Supplementation During Pregnancy and Test-Based Cognitive Performance at Age 10 Years: A Post Hoc Secondary Analysis of a Randomized Clinical Trial
Source: JAMA Netw Open. 2026 May 18;9(5):e2611464. doi: 10.1001/jamanetworkopen.2026.11464 (PMC13184775; doi:10.1001/jamanetworkopen.2026.11464)
Supplement: Supplement 3. — Data Sharing Statement [file jamanetwopen-e2611464-s003.pdf]

Data Sharing Statement

Frederiksen. High-Dose Vitamin D_3 Supplementation During Pregnancy and Test-Based Cognitive Performance at Age 10 Years. *JAMA Netw Open*. Published May 18, 2026. doi:10.1001/jamanetworkopen.2026.11464

Data
Additional Information: Clinicaltrials.gov NCT00856947
Data available: No

Additional Information
Explanation for why data not available: Individual-level personally identifiable clinical data from the children participating in the cohort cannot be made freely available, to protect the privacy of the participants and their families, in accordance with the Danish Data Protection Act and European Regulation 2016/679 of the European Parliament and of the Council (GDPR) that prohibit distribution even in pseudo-anonymized form. However, research collaborations are welcome, and data can be made available under a joint research collaboration by contacting COPSAC. Requests will be answered within two weeks. Data use is restricted to purposes within childhood health and disease.
